# Evolution of metabolite and volatile compounds in Chinese bayberry during juice processing, fermentation, and distillation

**DOI:** 10.1016/j.fochx.2026.104108

**Published:** 2026-06-15

**Authors:** Mostafa Saeed, Chaochao Zhou, Zhuyun Chen, Guoyun Wang, Ting Tu, Huimin Jia, Yun Jiao, Junbei Ni, Xian Li, Zhongshan Gao, Lan Zhao

**Affiliations:** a Institute of Fruit Science, College of Agriculture and Biotechnology, Zhejiang University, Hangzhou 310058, China; b Yuyao Agriculture Technology Extension Center, Ningbo, Yuyao 315400, China; c State Key Laboratory of Food Science and Resources, Nanchang Key Laboratory of Fruit and Vegetable Nutrition and Processing, Institute of Nutrition and School of Food Science, Nanchang University, Nanchang 330047, Jiangxi, China; d Department of Pomology, Faculty of Agriculture, Alexandria University, Alexandria 21545, Egypt; e Shunmei Breeding and Propagation Center for Chinese Bayberry, Yuyao, China; f College of Agronomy, Jiangxi Agricultural University, Nanchang 330045, China; g Institute of Forestry, Ningbo Academy of Agricultural Science, Ningbo, 315040, China; h Yuyao Southern Characteristic Fruit Tree Research Institute, Ningbo, 315040, China; i Yuyao Shunyi Yangberry Wine/Liquor Ltd, Yuyao 315400, Ningbo, China

**Keywords:** Chinese bayberry, Metabolome, Aroma, Odor activity, Fresh juice, Fermented juice, Distilled liquor

## Abstract

Chinese bayberry (*Morella rubra*) is an exotic fruit with a short postharvest shelf life, and its juice is valued for its distinctive aroma, making it suitable for producing characteristic liquor. Varietal differences and processing have a major effect on aroma, but systematic profiling of volatiles during production stages remains limited. In this study, we examined the aroma profiles of three bayberry varieties (‘Biqi’, ‘Dongkui’, and ‘Xiazhihong’) at three key stages: fresh juice, fermented juice, and distilled liquor. Using GC–MS analysis, terpenoids and esters had the most pronounced shifts during processing, with several compounds identified as key markers for each stage. Fermentation primarily altered terpenoid content, while distillation selectively enriched volatile esters. Odor activity analysis revealed that fruity and green notes dominated the fermented juice aroma, while woody and sweet descriptors prevailed in distilled liquor. ‘Biqi’ contained higher levels of several aroma-active constituents and received superior sensory scores. These findings reveal variety-specific volatile fingerprints and highlight how sequential processing shapes the aroma chemistry of bayberry liquor.

## Highlights

• Profiles of bayberry varieties as fresh and fermented juice, and distilled liquor.
• Terpenoids and esters were the dominant volatiles altered by processing.
• Fermentation altered terpenoids; distillation selectively enriched esters.
• Superior ‘Biqi’ liquor sensory scores linked to elevated terpenoid content.
• KEGG analysis linked terpenoid and ester pathways to aroma formation in bayberry.

## 1 Introduction

Chinese bayberry (*Morella rubra*) is a valuable fruit, renowned for its rich phytochemical composition, containing substantial amounts of flavonoids, phenolic acids, polysaccharides, organic acids, proteins, alkaloids, steroids, and various essential vitamins (Saeed et al., 2025). The economic importance of bayberry is underscored by its contribution to China's fruit industry, accounting for approximately 6.5% of the total economic value of fruit production nationwide (Yang et al., 2017). This commercial significance has led to expanding international trade, with fresh bayberry fruits and their processed products being exported to numerous overseas markets, including Singapore, Italy, Spain, and France (Tian et al., 2019). However, bayberry production faces significant postharvest challenges. The harvest season coincides with the hot and rainy period from June to July, making the fruit particularly vulnerable to mechanical damage and microbiological decay (Saeed, Zhao, Rashwan, et al., 2024). These postharvest issues substantially diminish the commercial value, with recent estimates indicating that more than 30% of the annual bayberry yield is lost to spoilage and deterioration, resulting in considerable economic impacts (Yang et al., 2023). These limitations have driven the development of processed bayberry products as a crucial strategy to reduce postharvest losses, extend market availability, and enhance added value.

Processing the fruit has seen significant expansion in recent years, with increasing yields prompting the development of diverse products including sweets, jams, juice drinks, canned products, dried snacks, and bayberry wines (Cao et al., 2020; Cheng, Chen, Li, et al., 2015). Bayberry juice processing has emerged as the most promising solution to reduce postharvest losses (Chen et al., 2022), but current industrial methods struggle to maintain the authentic flavor profile of processed bayberry juice, often failing to adequately preserve the characteristic taste and aroma of the fresh fruit (Liu, Chen, et al., 2023). An important consideration in bayberry processing is the significant variability among different cultivars. Research has demonstrated that various bayberry varieties have distinct colors, aroma profiles, taste, and chemical compositions (Cheng et al., 2016). These cultivar-specific differences extend to internal substance content, storage durability, and processing suitability, with direct implications for the sensory qualities of processed products (Tian et al., 2019). For instance, critical processing parameters such as terpenoids and ester components, which influence the final product's sensory attributes, vary significantly among cultivars (Cao et al., 2020). Surprisingly, despite these known variations, the specific effects of cultivar differences on the volatile flavor compounds in processed bayberry products, particularly wines, remain largely unexplored in scientific literature.

Flavor quality, particularly aroma profile, is one of the most critical determinants of consumer acceptance and commercial value in fruit products (Cheng, Chen, Li, et al., 2015). Bayberry flavor development involves complex biochemical pathways that generate a diverse array of volatile organic compounds, including esters, aldehydes, alcohols, ketones, acids, alkenes, and hydrocarbons (Chen et al., 2018). These compounds contribute unique sensory characteristics, with their combinations, concentrations, and synergistic interactions defining distinct profiles across cultivars and processing stages (Wang, Chen, et al., 2023). The aroma profile of bayberry products serves as a key quality indicator for both fresh and processed fruit, enabling cultivar differentiation and also directly determines the sensory quality and market value of bayberry wines (Mamede et al., 2005). As a primary driver of consumer preference, aroma also predicts perceived product quality and therefore commercial potential (Yu et al., 2020). The characteristic aroma of bayberry arises from the complex mixture of volatile compounds, predominantly terpenoids (e.g., caryophyllene), alcohols, aldehydes, esters, and acids (Fang et al., 2020). Studies have also identified terpenoids as typically the most abundant volatile class in bayberry, followed by alcohols, aldehydes, ketones, esters, and acids (Kang et al., 2012; Xu et al., 2014).

Despite increasing research on bayberry processing, the evolution of volatile and aroma-active compounds in processing stages remains poorly understood, and systematic comparisons of cultivars and processing steps are limited (Tian et al., 2024). In particular, how flavor-related metabolites are transformed from fresh juice to fermented juice and distilled liquor has not been comprehensively investigated. This study aimed to characterize the changes in volatile compounds and metabolite profiles during bayberry processing. Three widely cultivated and distinctive Chinese bayberry varieties (‘Biqi’, ‘Dongkui’, and ‘Xiazhihong’) were analyzed using GC–MS-based volatile profiling combined with multivariate statistical analysis. The objectives were to (1) examine dynamic changes in volatile composition during processing, (2) compare cultivar-specific aroma characteristics, and (3) identify key compounds contributing to flavor formation. The findings will provide insights into bayberry flavor development and support the optimization of processing strategies for high-quality bayberry beverages.

## 2 Materials and methods

### 2.1 Plant material

Fruits from three dominant bayberry (*Morella rubra*) varieties with different color phenotypes were selected for this study: ‘Biqi’ (BQ, black), ‘Dongkui’ (DK, red), and ‘Xiazhihong’ (XZH, pink). All sampled trees were of comparable age and cultivated in the same commercial orchard in Yuyao, Ningbo City, Zhejiang Province, China (30°09′34.4”N, 121°02′30.6″E), ensuring consistent soil conditions, climate, and agricultural management practices. This controlled growing environment allowed any observed differences in fruit composition to be attributed primarily to varietal differences. Fruits were hand-harvested at commercial maturity in June 2024. Following harvest, undamaged fruits of uniform size and color development from each cultivar were immediately placed in pre-chilled boxes and transported to the laboratory within 1–2 h. Upon arrival, samples were divided into two groups. The first group was used to analyze the fruit quality, while the second group was processed as fresh fruit juice (J), fermented juice (F), and distilled liquor (D).

### 2.2 Fruit quality analysis

Fruit color parameters were measured using a Hunter Lab Mini Scan XE Plus colorimeter (Hunter Associates Laboratory, Inc., Reston, VA) with four measurements taken per fruit for ten individual fruits, to give three-dimensional color coordinates: L* (lightness; 0–100 scale), a* (green-red axis; −60 to +60), and b* (blue-yellow axis; −60 to +60). From these values, chroma (C*) and hue angle (*h°*) were derived using the equations: C = (a*^2^ + b*^2^)^1^/^2^ and *h°* = tan^−1^(b*/a*) (Saeed et al., 2025). For comprehensive fruit characterization, we measured physical parameters of all ten samples, including individual fruit weight, longitudinal length, and equatorial diameter. Juice quality was assessed through total soluble solids (TSS) content using an Atago PAL-BXIACID digital refractometer (Atago Co., Tokyo, Japan) and titratable acidity (TA) using an Atago PAL-1 digital refractometer (Atago Co., Tokyo, Japan). The maturity index was subsequently calculated as the TSS/TA ratio (Saeed, Zhao, Chen, et al., 2024). Fruit firmness was determined using a TA-XT-plus texture analyzer (Stable Micro Systems, UK) equipped with a 5-mm cylindrical probe. Measurements were taken at two equatorial points on each of ten fruits, with a test speed of 1 mm/s and a penetration depth of 5 mm. Firmness values, expressed in Newtons (N), were given by the maximum force required for probe penetration (Saeed et al., 2025).

### 2.3 Determination of sugars and organic acids in fruits

For the determination of soluble sugars and organic acids, 1.0 g of ground fresh bayberry flesh was homogenized with 3 mL of 80% ethanol (*v*/v) in a 10 mL centrifuge tube, then incubated at 45 °C for 20 min as described by Ni et al. (2022). After centrifugation at 8000 ×*g* for 20 min, the supernatant was collected and the residue re-extracted twice under identical conditions. The combined supernatants were evaporated under vacuum at 45 °C until complete ethanol removal (approximately 4–5 h), and the residue was reconstituted in 1.0 mL ultrapure water and filtered through a 0.22 μm membrane filter before injection. Sugars and organic acids were quantified by high-performance liquid chromatography (HPLC) on an Agilent 1260 Infinity II system (Agilent Technologies, Santa Clara, CA, USA) as described by Saeed, Zhao, Chen, et al. (2024). An Ultimate® XB-NH2 column (4.6 mm × 250 mm, particle size 5 μm) was used for sugar analysis, with refractive index detection, an acetonitrile/water (75:25, *v*/v) isocratic mobile phase at 1.0 mL/min, 35 °C, and a 10 μL injection volume (run time: 25 min). Organic acids were separated on an ODS C18 column (4.6 mm × 250 mm, particle size 5 μm; Beckman, USA) with diode array detection at 210 nm, using a mobile phase of 0.1% phosphoric acid in water (A) and methanol (B) at 0.8 mL/min, with a linear gradient from 2% to 15% B over 15 min, held for 5 min, then re-equilibrated to initial conditions (30 °C; injection volume 10 μL). Quantification was using external calibration curves of authentic standards: sucrose, fructose, and glucose for sugars (R^2^ ≥ 0.999), and citric, malic, tartaric, and ascorbic acids for organic acids (R^2^ ≥ 0.998).

### 2.4 Liquor processing procedures

Bayberry fermented juice and distilled liquor were prepared according to the established Technical Code of Practice for Fruit Distilled Liquor Production in Zhejiang (ICS 67.160.10; T/ZNZ 236–2023), as reported previously (Chen et al., 2024). For each variety, a batch of 50 kg of fresh bayberries per biological replicate (three replicates per variety; 150 kg total per variety) was pitted, pulped, and juiced, yielding 35–38 kg of fresh juice per batch. The juice was supplemented with 10% (*w*/w) and thoroughly mixed before fermentation. Specialized yeast (0.02 kg per 100 kg raw material) was added, and the mixture was allowed to stand for 1 h before sealing the fermentation vessel with polyethylene film and maintaining it at 18–35 °C for 45–90 days, with manual stirring on days 3, 10, and 17 to ensure homogeneity. Distillation was using a stainless-steel pot still with direct steam injection, operating at atmospheric pressure with a steam pressure of 0.2–0.3 MPa and a distillation rate of approximately 2–3 L/min. Fractions were collected based on alcohol content by volume: ≥50% (high-grade liquor), 40–50% vol (mid-grade), and 10–40% vol (low-grade); distillates below 10% vol were discarded as tail fractions. The fractions were blended and aged in ceramic vats for ≥6 months. The final product quality was assessed based on visual clarity (absence of suspended solids or precipitation), a distinct bayberry aroma, and a balanced sensory profile (soft, sweet taste with a clean finish), meeting premium fruit spirit standards.

### 2.5 Metabolomic profiling and volatile compound analysis

#### 2.5.1 Sample preparation

For comprehensive metabolomic characterization, we analyzed three bayberry varieties in triplicate (*n* = 9 each for fresh fruit juice, fermented juice, and distilled liquor; 27 samples in total). The extraction and quantification were performed by MetWare Biotechnology Co., Ltd. (Wuhan, China; www.metware.cn), following established protocols (Zou et al., 2020). Samples were analyzed using two platforms: an Ultra-Performance Liquid Chromatography-Electrospray Ionization Tandem Mass Spectrometry (UPLC-ESI-MS/MS) system and a Headspace solid-phase microextraction-gas chromatography–mass spectrometry (HS-SPME-GC–MS) system (Yuan et al., 2022). For relative quantification, 70% methanol extracts served as internal standards for LC-MS. For GC–MS volatile analysis, 3-hexanone-2,2,4,4-d4 was used as the internal standard for quantification. A saturated NaCl solution was added to each headspace vial as a salting-out agent to enhance partitioning of volatile compounds and improve SPME extraction efficiency. Metabolite identification followed validated methods (Zhu et al., 2023).

#### 2.5.2 UPLC metabolomics profiling

Non-volatile metabolites were analyzed on a UPLC-ESI-MS/MS system consisting of an ExionLC™ AD UPLC (SCIEX, https://sciex.com.cn/) coupled to an Applied Biosystems 4500 Q TRAP mass spectrometer (SCIEX). Chromatographic separation was on an Agilent SB-C18 column (1.8 μm, 2.1 mm × 100 mm) maintained at 40 °C. The mobile phase consisted of 0.1% formic acid in ultrapure water (solvent A) and 0.1% formic acid in acetonitrile (solvent B), delivered at 0.35 mL/min with the following gradient: 95% A/5% B (initial), linearly transitioning to 5% A/95% B over 9 min, held for 1 min, returning to 95% A/5% B in 1.1 min, followed by 2.9 min re-equilibration; the injection volume was 4 μL. The ESI source was operated in both positive and negative ion modes with the following parameters: source temperature 550 °C; ion spray voltage +5500 V (positive) / −4500 V (negative); ion source gas I (GS1) 50 psi; ion source gas II (GS2) 60 psi; curtain gas (CUR) 25 psi; collision-activated dissociation set to high. Quantification was in multiple reaction monitoring (MRM) mode with nitrogen as the collision gas (medium setting); declustering potential and collision energy were individually optimized for each MRM transition, and specific MRM transition sets were monitored for each chromatographic period, corresponding to the elution windows of the target metabolites.

#### 2.5.3 GC–MS volatile compound profiling

After sampling, volatile organic compounds (VOCs) were extracted from the headspace using a 120 μm DVB/CWR/PDMS SPME Arrow (Agilent Technologies, Santa Clara, CA, USA). Desorption of the VOCs from the SPME Arrow coating was in the injection port of the GC apparatus (Model 8890; Agilent) at 250 °C for 5 min in splitless mode. The identification and quantification of VOCs was using an Agilent Model 8890 GC and a 7000D mass spectrometer (Agilent), equipped with a 30 m × 0.25 mm × 0.25 μm DB-5MS (5% phenyl-polymethylsiloxane) capillary column. Helium was used as the carrier gas at a linear velocity of 1.2 mL/min. The injector temperature was kept at 250 °C. The oven temperature was programmed to 40 °C (3.5 min), increasing at 10 °C/min to 100 °C, then at 7 °C/min to 180 °C, and at 25 °C/min to 280 °C, with a hold for 5 min. Mass spectra were recorded in electron impact ionization mode at 70 eV. The quadrupole mass detector, ion source, and transfer line temperatures were set at 150, 230, and 280 °C, respectively. VOCs were identified and quantified using Selected Ion Monitoring (SIM) mode, based on retention-time matching and the extraction of characteristic qualitative and quantitative ions (*m*/*z*) for each analyte.

#### 2.5.4 Metabolites comparisons, KEGG annotation, and enrichment analysis

Two analytical frameworks were used: a within-variety, stage-specific comparison tracking volatile and metabolite changes of fresh juice vs fermented juice, and fermented juice vs distilled liquor, and a cross-varietal comparison assessing variety-dependent compositional differences at the fermentation and distillation stages. For the two-group analysis, differential metabolites were identified using VIP (VIP > 1) and absolute Log_2_FC (Log_2_FC ≥ 1.0). For the multi-group analysis, differential metabolites were identified using VIP (VIP > 1) and *P*-values (*P* < 0.05, ANOVA). Identified metabolites were annotated using the KEGG Compound database (http://www.kegg.jp/kegg/compound/), and annotated metabolites were then mapped to the KEGG pathway database (http://www.kegg.jp/kegg/pathway.html). Pathways with significantly regulated metabolites were then fed into metabolite set enrichment analysis, and the hypergeometric test's *p*-values to determine their significance.

### 2.6 Sensory evaluation analysis of distilled liquor

The sensory evaluation of Chinese bayberry distilled liquor was conducted according to the GB/T 15038–2006 (General Analytical Methods for Wine and Fruit Wine) and GB/T 10345–2022 (Analytical Methods for Baijiu) standards as described by Chen et al. (2024). A trained panel of ten members, each with over three years of experience in distilled-spirit assessment, completed refresher training before evaluation. Training included calibration to characteristic bayberry aroma and taste descriptors, intensity scaling with reference standards, and consensus-building on descriptor definitions **(Table S1)**. Only panelists who demonstrated consistent scoring were selected. Quantitative Descriptive Analysis was used to assess appearance, aroma, taste, and typicality, each defined by standardized descriptors and rating anchors. Samples (20 mL) were served in a randomized, monadic sequence within three-digit-coding, covered ISO glasses under controlled sensory booth conditions. Each panelist performed duplicate evaluations on non-consecutive days (with a minimum 48-h interval between sessions) to confirm reproducibility. Final scores for each panelist were calculated as the average of the two session scores. According to Zhejiang University regulations, formal ethical approval was not required. Nonetheless, informed consent was obtained from all participants, and their rights, privacy, and freedom to withdraw at any time were fully respected throughout the study.

### 2.7 Statistical analysis

All experimental data were derived from three biological replicates and are presented as means ± standard deviation. Statistical analyses were conducted using SPSS Statistics (version 22, IBM Corp., Armonk, NY, USA), with one-way ANOVA to assess significant differences. For metabolomic data visualization, heat maps were generated with metabolite peak areas normalized using a z-score transformation to account for abundance variations. Principal component analysis (PCA) was using the R function prcomp (www.r-project.org). The hierarchical cluster analysis (HCA) results for samples and metabolites were presented as heatmaps with dendrograms, while Pearson correlation coefficients (PCC) between samples were calculated using the cor function in R and presented solely as heatmaps. Both HCA and PCC were carried out using the R package ComplexHeatmap. For HCA, normalized metabolite signal intensities (unit-variance scaling) were visualized as a color spectrum. The correlation between sensory evaluation and key volatile compounds from the terpenoid and ester groups was compared, and the correlation heat map drawn using the online platform ChiPlot (https://www.chiplot.online/dubble_matrices_correlation_heatmap.html).

## 3 Results

### 3.1 Fruit quality analyses of three bayberry varieties

Significant differences in the basic fruit quality parameters at harvest were observed among the three Chinese bayberry varieties ‘Biqi’, ‘Dongkui’, and ‘Xiazhihong’ **(Table S2)**. Their distinct physiochemical characteristics may influence their suitability for juice processing and fermentation. Fruit color differs in the three varieties. ‘Xiazhihong’ (pink) fruits had significantly (*p* < 0.05) higher L* (25.08 ± 2.11), a* (12.40 ± 1.90), b* (3.17 ± 0.97), and C* (12.60 ± 1.99) values compared to ‘Biqi’ (black) and ‘Dongkui’ (dark red), indicating a brighter colored fruit. ‘Biqi’ had the highest total anthocyanin content (0.94 ± 0.11 mg/g FW), significantly (*p* < 0.05) greater than ‘Dongkui’ (0.71 ± 0.09 mg/g FW) and ‘Xiazhihong’ (0.39 ± 0.13 mg/g FW), which related to color index values and aligned well with the color variation. ‘Dongkui’ had the highest TSS content (12.80 ± 0.61°Brix), significantly (p < 0.05) greater than both ‘Xiazhihong’ (11.27 ± 0.49°Brix) and ‘Biqi’ (9.95 ± 1.01°Brix). However, ‘Xiazhihong’ had the highest TA value (1.55 ± 0.19%) and consequently the lowest TSS/TA ratio (7.27) compared to ‘Biqi’ (10.39) and ‘Dongkui’ (10.58).

Sucrose content was highest in ‘Dongkui’ (73.75 ± 3.53 mg/g FW), significantly (p < 0.05) exceeding both ‘Biqi’ (59.12 ± 1.92 mg/g FW) and ‘Xiazhihong’ (57.32 ± 1.61 mg/g FW). There were no significant differences in fructose and glucose levels in the three varieties. For organic acids, ‘Xiazhihong’ had the highest citric acid content (11.45 ± 0.35 mg/g FW), while ‘Dongkui’ had the highest malic (0.89 ± 0.11 mg/g FW) and tartaric acid (0.31 ± 0.08 mg/g FW). Ascorbic acid content was similar in ‘Biqi’ (0.15 ± 0.03 mg/g FW) and ‘Xiazhihong’ (0.17 ± 0.01 mg/g FW), both significantly (p < 0.05) higher than ‘Dongkui’ (0.11 ± 0.02 mg/g FW). These results demonstrate substantial varietal differences in fruit quality parameters that may significantly impact juice characteristics and fermentation outcomes. The higher sugar content in ‘Dongkui’, greater acidity in ‘Xiazhihong’, and elevated anthocyanin levels in ‘Biqi’ suggest each variety may have different metabolite profiles, especially related to aromatic and volatile active compounds. The physical differences, particularly in fruit size and firmness, may also influence juice extraction and yield.

### 3.2 Changes in sugar and acid compounds during processing of the three Chinese bayberry varieties

Compositional analysis of organic acids and sugars in fresh juice, fermented juice, and distilled liquor of three Chinese bayberry varieties revealed significant treatment-induced transformations **(Table S3)**. Fermentation and subsequent distillation led to a general decrease in the original acids present in fresh fruit juice, while an increase in some acids occurred during fermentation. Notably, seven organic acids were absent in fresh juice but detected in both fermented juice and distilled liquor: heptanoic acid, hexanoic acid, octanoic acid, 2-octanoic acid, valproic acid, p-tolyacetic acid, and 4-methoxybenzoic acid. This indicates their formation as fermentation byproducts or thermal derivatives. In general, the increase in these acids was greater during distillation than during fermentation, suggesting either continued chemical transformations or concentration effects associated with thermal processing. One exception was 2-octanoic acid, which decreased in the distilled liquor compared with the fermented juice across all varieties. Additionally, a notable decrease in 4-methoxybenzoic acid was recorded in the distilled liquor of the ‘Biqi’ variety compared to its fermented counterpart, pointing to potential varietal differences in acid stability or distillation dynamics.

There were distinct patterns of sugar metabolism transformation during Chinese bayberry processing **(Table S3)**. Seven key sugar metabolites were identified as central to the compositional shifts in fresh juice, fermented juice, and distilled liquor. Most were significantly downregulated during fermentation but subsequently increased during distillation, highlighting treatment-specific metabolic dynamics. Disaccharides, such as maltose and sucrose, decreased sharply during fermentation, between 85% to 99%, reflecting their rapid utilization by yeast. In contrast, monosaccharides, including fructose, glucose, and mannose, followed a biphasic trend, initially decreasing during fermentation and then increasing post-distillation. This pattern suggests intense microbial consumption during fermentation, followed by potential polysaccharide hydrolysis or sugar liberation during distillation. Ribose was the only sugar with a consistent decline throughout processing across all varieties (*p* < 0.01), suggesting limited stability under processing conditions. Varietal differences were especially pronounced in sugar profiles. These metabolic transformations underscore the dual influence of universal biochemical mechanisms and varietal specificity in shaping the chemical profile of bayberry-derived products. The pronounced acid accumulation and dynamic sugar interconversions are likely pivotal in modulating flavor development and overall beverage quality. Our findings provide a foundation for refining processing strategies to optimize sensory and nutritional attributes in bayberry-based wines and spirits.

### 3.3 Analysis of aroma-active compounds in fermented and fresh juice

Metabolome analysis of the different samples (fresh juice, fermented juice, and distilled liquor) was used to identify differences in aromatic and volatile compounds in the three bayberry varieties. Representative total-ion chromatograms for both LC-MS and GC–MS platforms, shown in **Figs. S1 and S2**, demonstrate consistent baseline stability, reproducible retention times, and high signal quality across all samples. The reliability of metabolite detection and quantification is further confirmed by multiple reaction monitoring, multi-peak chromatograms and integration peak reproducibility for randomly selected metabolites in all sample groups. Principal Component Analysis (PCA) further confirmed the distinct metabolic signatures of fresh and fermented juices **(Fig. S3A—C)**. PC1 clearly separated fermented samples across all varieties, explaining 86–88% of the variance. Those of ‘Biqi’ (BQ-F) clustered tightly along PC1 (86.46%), as did ‘Dongkui’ (DK-F; PC1: 87.65%) and ‘Xiazhihong’ fermented samples (XZH-F; PC1: 86.26%). This separation was primarily driven by terpenoids and esters, underscoring their role as discriminant biomarkers.

Volcano plot analysis highlighted extensive metabolic reprogramming **(Fig. S3D—F)**. ‘BQ-F' had 802 upregulated and 797 downregulated metabolites, while ‘DK-F' had 779 upregulated and 878 downregulated metabolites, and ‘XZH-F' had 803 upregulated and 767 downregulated. The comparative analysis of fresh and fermented juices from three bayberry varieties revealed significant alterations in metabolite composition driven by fermentation. Terpenoids, esters, and heterocyclic compounds constituted the dominant chemical classes in all varieties. In ‘Biqi’, the most abundant categories affected by fermentation were terpenoids (17%), followed by esters (10.5%) and heterocycle compounds (9%) **(Fig. S3G)**. The same trend was found in ‘Dongkui’ with different percentages: terpenoids (23.4%), esters (17.4%), and heterocycle compounds (14.2%) **(Fig. S3H)**, and similarly for ‘Xiazhihong’: terpenoids 24.1%, esters 17.3%, and heterocyclic compounds 13.6% **(Fig. S3I)**.

Metabolomic profiling revealed distinct clustering patterns in fresh and fermented juices across all three varieties **(Fig. S4A—C)**, with pronounced shifts in terpenoids, esters, and heterocyclic compounds, which align well with PCA, volcano plot, and alteration percentage. In ‘Biqi’, ‘BQ-F' had elevated *Z*-scores (>1.5) for terpenoids, esters, and heterocyclic compounds, while fresh juice (BQ-J) was enriched in amino acids and flavonoids (Z-score: 1.2–1.8). Similarly, there was marked upregulation of terpenoids, esters, and heterocyclic compounds (Z-score: 1.4–1.8) in ‘DK-F', contrasting with fresh juice samples (DK-J), where flavonoids, amino acids, and phenolic acids dominated (Z-score: 1.1–1.5). ‘Xiazhihong’ had a unique profile, with ‘XZH-F' accumulating terpenoids, esters, and heterocyclic compounds, as with ‘BQ-J' and ‘DK-J'. In contrast, fresh juice accumulates lignans and coumarins (Z-score: 1.7), as well as amino and organic acids (Z-score: 1.2–1.6). These findings underscore the role of fermentation in modulating varietal-specific metabolite signatures. KEGG pathway analysis identified key metabolic pathways influenced by fermentation (Rich Factor > 0.8, *p*-value <0.05). In both ‘Biqi’ and ‘Dongkui’ **(Fig. S4D, E)**, sesquiterpenoid and triterpenoid biosynthesis were significantly enriched, aligning with observed terpenoid alteration by fermentation. ‘XZH-F' activated monoterpenoid biosynthesis, corroborating the Z-score rise in terpenoid derivatives **(Fig. S4F)**. Generally, terpenoid derivative biosynthesis was a shared pathway across the three varieties, highlighting the alteration in terpenoids during fermentation.

Fermentation induced significant alterations in the volatile and aromatic profiles of all three bayberry varieties, with distinct patterns of change observed for different chemical classes **(**Table 1**)**. The most pronounced changes occurred in terpenoid compounds, with the accumulation of α-phellandrene-8-ol being detected in fermented juice in the three varieties, whereas this was absent in fresh juice. In contrast, β-Gurjunene and Carveol II were absent in the fermented juice but were accumulated in the fresh juice of the three varieties. β-caryophyllene, one of the common terpenoids in bayberry fruits, is already high in the fresh juice of ‘Biqi’ (10.48%) and ‘Dongkui’ (11.65%) compared to ‘Xiazhihong’ (1.74%). However, fermentation downregulated the content of β-caryophyllene in all three varieties. α-Humulene levels in ‘Xiazhihong’ differed from those in both ‘Biqi’ and ‘Dongkui’, as it was upregulated from 0.12% (XZH-J) to 1.09% (XZH-F), while it was downregulated in ‘BQ-F' and ‘DK-F'.

**Table 1:** Key differential volatile components of fermented and fresh juice of three Chinese bayberry varieties.

Category	Components	Formula	CAS	NIST_RI	Odor Character	Threshold	Relative content %		*p-*value	Type	Relative content %		*p-*value	Type	Relative content %		p-value	Type
							**BQ-F**	**BQ-J**			**DK-F**	**DK-J**			**XZH-F**	**XZH-J**		
Terpenoids	α-Phellandrene-8-ol	C_3_H_6_N_2_O_2_	1686-20-0	1167	–	–	2.41 ± 0.05	–	0.000	Up	2.22 ± 0.15	–	0.001	Up	2.24 ± 0.11	–	0.000	Up
	*α*-Humulene	C_15_H_24_	6753-98-6	1454	woody	1.60E-01	1.39 ± 0.09	2.48 ± 0.15	0.022	Down	0.86 ± 0.07	2.73 ± 0.26	0.024	Down	1.09 ± 0.11	0.12 ± 0.01	0.022	Up
	*β*-Caryophyllene	C_15_H_24_	87–44–5	1419	sweet, woody, spice, clove	1.54E+00	0.04 ± 0.00	10.48 ± 0.13	0.009	Down	0.07 ± 0.01	11.65 ± 0.15	0.011	Down	0.02 ± 0.00	1.74 ± 0.10	0.009	Down
	β-Gurjunene	C_15_H_24_	17,334–55-3	1432	–	–	–	8.86 ± 0.06	0.011	Down	–	9.81 ± 0.09	0.010	Down	–	1.16 ± 0.09	0.011	Down
	Carveol II	C_10_H_16_O	1197-06-4	1213.5	–	–	–	1.68 ± 0.18	0.001	Down	–	1.11 ± 0.18	0.001	Down	–	3.33 ± 0.24	0.001	Down
Heterocyclic compounds	3-Acetyl-2-oxo-1,3-oxazolidine	C_5_H_7_NO_3_	1432-43-5	1174	–	–	14.95 ± 0.47	–	0.002	Up	8.26 ± 0.12	–	0.002	Up	14.59 ± 0.24	–	0.002	Up
	4-Acetylpyrazole	C_5_H_6_N_2_O	25,016–16-4	1156	–	–	1.76 ± 0.05	0.72 ± 0.04	0.001	Up	1.73 ± 0.09	0.49 ± 0.07	0.004	Up	1.60 ± 0.06	1.33 ± 0.14	0.001	Up
	2-Furanacrolein	C_7_H_6_O_2_	623–30-3	1111	green, fruity, spicy, woody	1.31E+01	1.37 ± 0.01	–	0.002	Up	2.51 ± 0.09	–	0.003	Up	1.77 ± 0.01	–	0.002	Up
	Pyrazine, 2-isobutyl-3-methoxy	C_9_H_14_N_2_O	24,683–00-9	1181	green bell pepper, pea	2.00E-06	–	4.06 ± 0.39	0.001	Down	–	2.68 ± 0.41	0.002	Down	–	7.77 ± 0.63	0.001	Down
Ketone	Cashmeran	C_14_H_22_O	33,704–61-9	1508	woody, rich, spicy, musky,	–	2.19 ± 1.01	–	0.004	Up	2.98 ± 0.20	–	0.000	Up	2.58 ± 0.14	–	0.004	Up
	3-Hexanone, 1-phenyl-	C_12_H_16_O	29,898–25-7	1427	–	–	0.01 ± 0.00	8.24 ± 0.11	0.009	Down	0.03 ± 0.00	9.46 ± 0.23	0.013	Down	0.01 ± 0.00	1.34 ± 0.08	0.009	Down
Ester	3,4,5,6-2H4-Methyl 2-Hydroxybenzoate	HOC_6_D_4_COOCH_3_	1,219,802–12-6	1196.934460887949	–	–	11.44 ± 0.31	–	0.000	Up	11.41 ± 0.31	–	0.000	Up	11.03 ± 0.42	–	0.000	Up
Hydrocarbons	2-Methyl-7-*exo*-vinylbicyclo[4.2.0]oct-1(2)-ene	C_11_H_16_	107,914–89-6	1112	–	–	13.85 ± 0.11	–	0.003	Up	26.31 ± 1.47	–	0.006	Up	17.79 ± 0.34	–	0.003	Up

BQ-F: ‘Biqi’ fermented juice, DK-F: ‘Dongkui’ fermented juice, and XZH-F: ‘Xiazhihong’ fermented juice. BQ-J: ‘Biqi’ fresh juice, DK-J: ‘Dongkui’ fresh juice, and XZH-J: ‘Xiazhihong’ fresh juice. NIST_RI: NIST Retention Index. Up/Down refers to the upregulation/downregulation in fermented juice (F) relative to fresh juice (J). The symbol ‘-’ denotes that the parameter was not detected or not reported. Volatile compounds were analyzed and identified using HS-SPME-GC–MS.

Two heterocyclic compounds, 3-Acetyl-2-oxo-1,3-oxazolidine and 2-Furanacrolein, were present in fermented juice and absent in fresh juice in all varieties. Notably, 4-acetylpyrazole was upregulated, while 2-isobutyl-3-methoxy pyrazine was downregulated in fermented juice compared with fresh juice in the three varieties. We observed compounds from different metabolite categories, such as cashmeran (ketone), 3,4,5,6-2H4-Methyl 2-Hydroxybenzoate (ester), and 2-Methyl-7-*exo*-vinylbicyclo [4.2.0] oct-1(2)-ene (hydrocarbons), which were present in fermented juice and absent in the fresh juice of the three varieties. The observed changes in volatile composition suggest that fermentation induces universal and variety-specific transformations in bayberry juices. The consistent increase in heterocyclic compounds, esters, acids, and hydrocarbons, along with alterations in terpenoids in all varieties, reflects intrinsic biochemical differences in the juice-processing stages and bayberry varieties. These findings have important implications for understanding how fermentation modifies the flavor and aromatic profiles of bayberry juices.

### 3.4 Characteristics of aroma compounds in fermented juice and distilled liquor

**Fig. 1:**
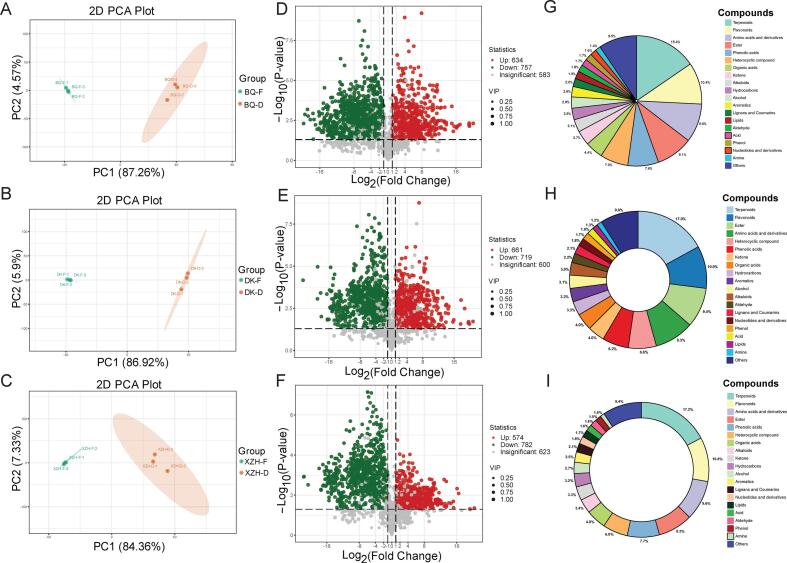
Metabolomics analysis overview comparing fermented juice and distilled liquor for three bayberry varieties. Principal component analysis (PCA) for ‘Biqi’ (A), ‘Dongkui’ (B), and ‘Xiazhihong’ (C). BQ-F: ‘Biqi’ fermented juice, DK-F: ‘Dongkui’ fermented juice, and XZH-F: ‘Xiazhihong’ fermented juice. BQ-D: ‘Biqi’ distilled liquor, DK-D: ‘Dongkui’ distilled liquor, and XZH-D: ‘Xiazhihong’ distilled liquor. Volcano plots comparing differentially accumulating metabolites (DAMs) for ‘Biqi’ (D), ‘Dongkui’ (E), and ‘Xiazhihong’ (F). The Y-axis indicates Log10 (*P*-value), and the X-axis indicates Log2 fold change. Significantly upregulated and downregulated metabolites are highlighted in red and green, respectively, while nonsignificant compounds are shown in grey. Pie charts, G-I, display the proportional changes in metabolite classes for ‘Biqi’, ‘Dongkui’, and ‘Xiazhihong’, respectively. (For interpretation of the references to color in this figure legend, the reader is referred to the web version of this article.)

The transition from fermented juice to distilled liquor induced significant metabolic reorganization in all three Chinese bayberry varieties, as demonstrated by multivariate analysis and compositional profiling **(**Fig. 1**)**. PCA revealed a clear separation between fermented and distilled products, with PC1 explaining 84.36–87.26% of variance **(**Fig. 1A-C**)**. The distilled liquors formed tight clusters distinct from their fermented counterparts, with ‘Xiazhihong’ having the greatest dispersion (PC2: 7.33%), suggesting variety-specific responses to distillation. Notably, ‘Biqi’ samples had the most pronounced separation (PC1: 87.26%), indicating particularly robust metabolic changes during distillation. Volcano plot analysis identified 634, 661, and 574 upregulated and 757, 719, and 782 downregulated metabolites during distillation for ‘Biqi’, ‘Dongkui’, and ‘Xiazhihong’, respectively **(**Fig. 1D-F**)**. Terpenoids (15.4–17.2%) and flavonoids (10.0–10.6%) emerged as the dominant chemical classes in distilled products **(**Fig. 1G-I**)**. The distillation process consistently altered the amino acid content (9.3–9.6%) and also the ester content (8.3–9.3%) across all varieties.

**Fig. 2:**
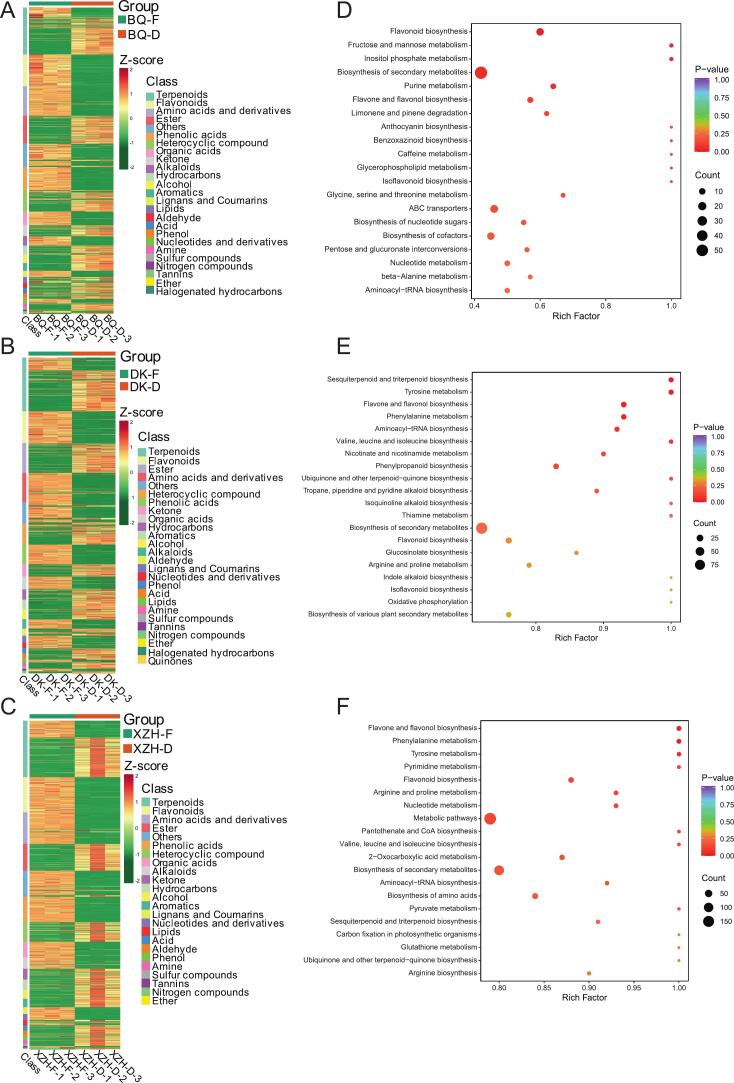
Heat maps and KEGG-enriched pathways of the detected metabolites in the three bayberry varieties during juice processing stages (fermented juice vs distilled liquor). Heat map analysis of metabolites of fermented juice vs distilled liquor for ‘Biqi’ (A), ‘Dongkui’ (B), and ‘Xiazhihong’ (C), with color indicating metabolite accumulation level, ranging from low (green) to high (red). The top 20 most enriched KEGG pathways of the DAMs between fermented and distilled liquor for ‘Biqi’ (D), ‘Dongkui’ (E), and ‘Xiazhihong’ (F). (For interpretation of the references to color in this figure legend, the reader is referred to the web version of this article.)

This process induced significant metabolic reorganization in processed products of all three Chinese bayberry varieties, as evidenced by comprehensive *Z*-score and pathway analyses **(**Fig. 2**)**. Metabolite heat map analysis revealed that distillation universally activated terpenoids, esters, and heterocyclic compounds that accumulate in the three varieties **(**Fig. 2A-C**)**. The patterns of flavonoids, phenolic acids, and amino acids were upregulated in fermented juice compared to distilled liquor. Pathway enrichment analysis demonstrated variety-specific metabolic shifts between fermentation and distillation processing. ‘BQ-D' had significant enrichment in flavonoid biosynthesis pathways (Rich Factor = 0.95), particularly flavone and flavonols metabolism. ‘DK-D' had unique activation of sesquiterpenoid and triterpenoid biosynthesis (Rich Factor = 0.98), while ‘Xiazhihong’ had a unique alteration of flavone and flavonols biosynthesis and phenylalanine metabolism **(**Fig. 2D-F**)**. The distillation process amplified existing varietal differences while introducing new compositional signatures. These results demonstrate that while distillation induces characteristic metabolic shifts across all bayberry varieties, their unique biochemical fingerprints led to distinct aromatic and functional profiles in the final distilled products, with implications for flavor optimization and bioactive compound retention.

The key differential volatile components between fermented juice and distilled liquor are shown in Table 2. The most dramatic changes were in esters, with a remarkable accumulation of long-chain ethyl esters in distilled products. Ethyl caprate increased from negligible levels (0.01–0.16%) in fermented juice to 12.58 ± 0.84% (BQ-D), 13.49 ± 1.01% (DK-D), and 14.72 ± 2.25% (XZH-D) in distilled liquors, demonstrating active esterification during distillation. Conversely, the opposite was true for methyl 2-hydroxybenzoate and isobornyl formate, which were lower in distilled liquor than in fermented juice, suggesting differential stability of ester compounds during processing. Terpenoid profiles had complex transformation patterns. While most terpenoids, such as norbornane and α-curcumene, decreased significantly during distillation, oxygenated terpenes such as 3-pinanol increased substantially from 0.10 ± 0.01% to 1.35 ± 0.09% in ‘BQ-D', from 0.12 ± 0.01% to 1.59 ± 0.15% in ‘DK-D', and from 0.14 ± 0.01% to 1.45 ± 0.15% in ‘XZH-D'. This selective preservation of oxygenated forms was consistent across all varieties, suggesting greater stability or increased formation during distillation.

**Table 2:** Key differential volatile components of fermented juice and distilled liquor of three Chinese bayberry varieties.

Category	Components	Formula	CAS	NIST_RI	Odor Character	Threshold	Relative content %		*p-*value	Type	Relative content %		*p-*value	Type	Relative content %		*p-*value	Type
							**BQ-F**	**BQ-D**			**DK-F**	**DK-D**			**XZH-F**	**XZH-D**		
Esters	3,4,5,6-2H4-Methyl 2-Hydroxybenzoate	HOC_6_D_4_COOCH_3_	1,219,802–12-6	1196.934460887949	–	–	11.44 ± 0.31	0.42 ± 0.03	0.000	UP	11.41 ± 0.31	0.44 ± 0.07	0.000	Up	11.03 ± 0.42	0.62 ± 0.03	0.000	Up
	Isobornyl formate	C_11_H_18_O_2_	1200-67-5	1233	woody, musty	–	1.90 ± 0.06	0.06 ± 0.00	0.000	Up	1.85 ± 0.08	0.06 ± 0.01	0.000	Up	1.81 ± 0.07	0.09 ± 0.00	0.000	Up
	Ethyl caprate	C_12_H_24_O_2_	110–38-3	1398	sweet, waxy, fruity	5.00E-03	0.01 ± 0.00	12.58 ± 0.84	0.000	Down	0.16 ± 0.10	13.49 ± 1.01	0.000	Down	0.03 ± 0.01	14.72 ± 2.25	0.003	Down
	Dodecanoic acid, ethyl ester	C_14_H_28_O_2_	106–33-2	1594	sweet, waxy, floral	5.00E-01	–	2.82 ± 0.27	0.000	Down	0.02 ± 0.02	2.85 ± 0.12	0.001	Down	–	2.60 ± 0.50	0.026	Down
	Formic acid butyl ester	C_5_H_10_O_2_	592–84-7	724	fruity	3.70E-01	–	4.86 ± 0.17	0.005	Down	–	4.83 ± 0.74	0.023	Down	–	6.40 ± 1.18	0.049	Down
	Ethyl 9-decenoate	C_12_H_22_O_2_	67,233–91-4	1388	fruity	2.00E-01	0.01 ± 0.00	11.75 ± 0.84	0.000	Down	0.15 ± 0.09	12.46 ± 0.98	0.000	Down	0.03 ± 0.01	13.78 ± 2.07	0.003	Down
Terpenoids	Norbornane	C_15_H_24_	25,532–78-9	1448	–	–	3.21 ± 0.28	0.02 ± 0.00	0.005	Up	2.21 ± 0.20	0.02 ± 0.00	0.012	Up	2.61 ± 0.42	0.01 ± 0.00	0.025	Up
	p-Mentha-1,5-dien-8-ol	C_10_H_16_O	1686-20-0	1167	–	–	2.41 ± 0.05	0.04 ± 0.01	0.000	Up	2.22 ± 0.15	0.04 ± 0.01	0.000	Up	2.24 ± 0.11	0.06 ± 0.01	0.000	Up
	α-Curcumene	C_15_H_22_	644–30-4	1483	herbal	–	1.98 ± 0.05	0.09 ± 0.00	0.000	Up	1.89 ± 0.08	0.14 ± 0.01	0.000	Up	1.86 ± 0.09	0.06 ± 0.00	0.000	Up
	p-Menth-8-en-1-ol, stereoisomer	C_10_H_18_O	7299-40-3	1161	–	–	1.85 ± 0.04	0.05 ± 0.01	0.000	Up	1.76 ± 0.10	0.03 ± 0.01	0.000	Up	1.74 ± 0.07	0.02 ± 0.01	0.000	Up
	3-Pinanol	C_10_H_18_O	27,779–29-9	1179	–	–	0.10 ± 0.00	1.35 ± 0.09	0.000	Down	0.12 ± 0.00	1.59 ± 0.15	0.000	Down	0.14 ± 0.01	1.45 ± 0.15	0.005	Down
Hydrocarbons	2-Methyl-7-exo-vinylbicyclo[4.2.0]oct-1(2)-ene	C_11_H_16_	107,914–89-6	1112	–	–	13.85 ± 0.11	0.32 ± 0.09	0.000	Up	26.31 ± 1.47	0.93 ± 0.24	0.006	Up	17.79 ± 0.34	0.76 ± 0.16	0.000	Up
	1,5-Cycloundecadiene, 8,8-dimethyl-9-methylene-	C_14_H_22_	62,338–54-9	1485	–	–	2.38 ± 0.07	0.09 ± 0.00	0.000	Up	2.27 ± 0.10	0.13 ± 0.01	0.000	Up	2.24 ± 0.12	0.07 ± 0.01	0.000	Up
	Undecane, 2-methyl-	C_12_H_26_	7045-71-8	1164	–	1.00E+01	1.55 ± 0.03	0.02 ± 0.00	0.000	Up	1.47 ± 0.08	0.02 ± 0.00	0.000	Up	1.45 ± 0.05	0.02 ± 0.01	0.000	Up
	Nonane, 3-methyl-5-propyl-	C_13_H_28_	31,081–18-2	1185	–	–	0.05 ± 0.01	1.10 ± 0.08	0.000	Down	0.07 ± 0.00	1.26 ± 0.11	0.000	Down	0.09 ± 0.01	1.20 ± 0.13	0.004	Down
Heterocyclic compounds	2-Hexanoylfuran	C_10_H_14_O_2_	14,360–50-0	1239	sweet, fruity, green	–	2.68 ± 0.09	0.08 ± 0.01	0.000	Up	2.61 ± 0.12	0.09 ± 0.01	0.000	Up	2.56 ± 0.10	0.12 ± 0.01	0.000	Up
	5,6,7,8-Tetrahydroquinoxaline	C_8_H_10_N_2_	34,413–35-9	1212	musty, roasted	–	2.27 ± 0.16	0.05 ± 0.01	0.005	Up	1.66 ± 0.09	0.06 ± 0.00	0.001	Up	1.68 ± 0.32	0.08 ± 0.01	0.036	Up
	Ethanone, 1-(1H-pyrazol-4-yl)-	C_5_H_6_N_2_O	25,016–16-4	1156	–	–	1.76 ± 0.05	0.07 ± 0.00	0.000	Up	1.73 ± 0.09	0.07 ± 0.01	0.000	Up	1.60 ± 0.06	0.15 ± 0.01	0.002	Up
	2-Propenal, 3-(2-furanyl)	C_7_H_6_O_2_	623–30-3	1111	woody, green, fruity	1.31E+01	1.37 ± 0.01	0.03 ± 0.01	0.000	Up	2.51 ± 0.09	0.08 ± 0.02	0.003	Up	1.77 ± 0.01	0.07 ± 0.01	0.000	Up
Alcohol	Benzenemethanol, α.-2-propenyl-	C_10_H_12_O	936–58-3	1244	–	–	1.20 ± 0.04	0.04 ± 0.00	0.000	Up	1.17 ± 0.05	0.04 ± 0.01	0.000	Up	1.15 ± 0.05	0.06 ± 0.00	0.000	Up
Phenol	1,3-Benzenediol, 4,5-dimethyl-	C_8_H_10_O_2_	527–55-9	1490	–	–	3.16 ± 0.08	0.04 ± 0.00	0.000	Up	3.02 ± 0.11	0.05 ± 0.01	0.000	Up	2.96 ± 0.11	0.05 ± 0.00	0.000	Up

BQ-F: ‘Biqi’ fermented juice, DK-F: ‘Dongkui’ fermented juice, and XZH-F: ‘Xiazhihong’ fermented juice. BQ-D: ‘Biqi’ distilled liquor, DK-D: ‘Dongkui’ distilled liquor, and XZH-D: ‘Xiazhihong’ distilled liquor. NIST_RI: NIST Retention Index. Up/Down refers to the upregulation/downregulation in fermented juice (F) relative to distilled liquor (D). The symbol ‘-’ denotes that the parameter was not detected or not reported. Volatile compounds were analyzed and identified using HS-SPME-GC–MS.

Hydrocarbons showed similar behavior, with 2-hexanoylfuran decreasing from 2.68 ± 0.09% to 0.08 ± 0.01% in ‘BQ-D', from 2.61 ± 0.12% to 0.09 ± 0.01% in ‘DK-D', and from 2.56 ± 0.10% to 0.12 ± 0.01% in ‘XZH-D'. Variety-specific patterns emerged in alcohol and phenolic compounds. Phenolic compounds decreased in all cases: 1,3-benzenediol dropped from approximately 3% in fermented juices to 0.045% in distilled products, with 1,3-benzenediol, 4,5-dimethyl (alcohol) following the same trend. These results demonstrate that distillation induces both universal and variety-specific transformations in bayberry volatile profiles. The process favors the formation and preservation of certain compound classes (esters) while degrading others (terpenoids). The consistent patterns across varieties suggest fundamental chemical transformations during distillation.

### 3.5 Comparison of aroma compounds in the fermented juice of the three bayberry varieties

**Fig. 3:**
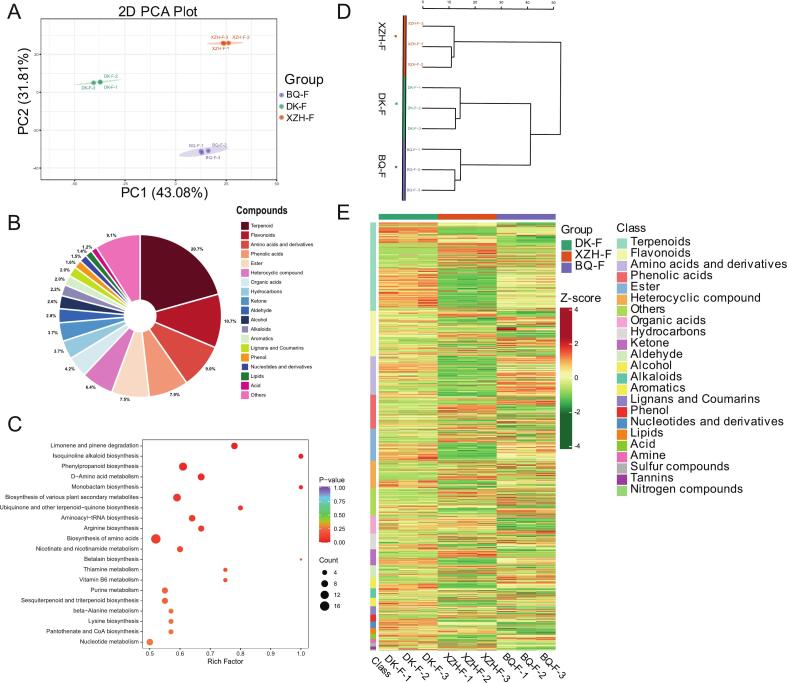
Overview of metabolites and aroma compounds comparing three bayberry varieties (‘Biqi’, ‘Dongkui’, and ‘Xiazhihong’) at the fermented juice stage. (A) Principal component analysis (PCA). (B) Pie chart depicting the percentage of alteration in metabolite categories. (C) KEGG enrichment analysis. (D) Hierarchical clustering analysis of the three varieties. (E) Heatmap for three bayberry varieties, with color gradients representing relative accumulation levels (green: low; red: high). (For interpretation of the references to color in this figure legend, the reader is referred to the web version of this article.)

Metabolomic analysis was used to identify volatile compounds in fermented juices from the three Chinese bayberry varieties. There were significant varietal differences in the metabolomic profiles of fermented Chinese bayberry juice, highlighting distinct compositional profiles and metabolic pathway activities **(**Fig. 3**)**. There was a clear separation of PCA in the varieties, with PC1 explaining 43.08% of the variance, indicating fundamental biochemical differences in the fermented products **(**Fig. 3A**)**. The PCA plot had tight clustering within each variety group, indicating consistent metabolic signatures for each cultivar. Terpenoids (20.7%), flavonoids (10.7%), amino acids (9%), phenolic acids (7.9%), esters (7.5%), and heterocyclic compounds (6.4%) were the dominant chemical classes, which differed across all varieties. Other metabolite classes, including ketones (3.7%), aldehydes (2.8%), alcohols (2.6%), and acids (1.2%), even with lower alteration percentages, are also important because they are closely related to the aromatic and volatile active compounds **(**Fig. 3B**)**.

Pathway enrichment analysis revealed variety-specific metabolic patterns: limonene and pinene degradation, as well as ubiquinone and other terpenoid-quinone biosynthesis, showed significant activation with a high rich factor and metabolite count **(**Fig. 3C**)**. Cluster analysis separated the varieties into three distinct groups, with greater metabolic similarity of ‘BQ-F' and ‘DK-F' compared to ‘XZH-F', demonstrating that the content of the fermented juice for ‘Biqi’ is more similar to ‘Dongkui’ than ‘Xiazhihong’ **(**Fig. 3D**).** Heatmap of the three varieties identified terpenoids as the dominant chemical class across all varieties **(**Fig. 3E**)**, with ‘DK-F' having the highest levels (*Z*-score: 2.8). There was significant variation in flavonoids, with the highest accumulation in ‘BQ-F' (Z-score: 2.1) compared to ‘DK-F' (1.8) and ‘XZH-F' (1.5). Amino acid derivatives were particularly enriched in ‘BQ-F' (Z-score: 2.3), while phenolic acids had similar levels to ‘XZH-F' (Z-scores: 1.8–2.3). Ester content was highest in ‘DK-F' (Z-score: 1.9), potentially contributing to its distinctive aromatic profile. These biochemical differences likely contribute to the distinct sensory and functional properties of each variety's fermented product, suggesting potential for targeted applications in functional beverage development. The consistent patterns observed within varieties, but the clear differentiation between them, underscore the importance of variety selection in bayberry fermentation processes.

The top differential volatile components in the fermented juices of three Chinese bayberry varieties had notable differences in terpene and ester profiles, which are key contributors to aroma and flavor characteristics **(Table S4)**. Among the terpenoids, α-humulene and α-curcumene were the most abundant in all three varieties. α-Humulene was highest in ‘BQ-F' (1.39 ± 0.09%), followed by ‘XZH-F' (1.09 ± 0.11%) and ‘DK-F' (0.86 ± 0.07%). Similarly, α-curcumene was comparably high in all samples: in ‘BQ-F' (1.98 ± 0.05%), ‘DK-F' (1.89 ± 0.08%), and in ‘XZH-F' (1.86 ± 0.09%). Levels of β-Caryophyllene were low in all three, with DK-F having the highest content (0.07 ± 0.01%). Notably, the level of caryophyllenyl alcohol was substantial in ‘BQ-F' (0.20 ± 0.02%) and ‘DK-F' (0.22 ± 0.01%) but markedly reduced in ‘XZH-F' (0.01 ± 0.00%). Both cedrene and β-cedrene were detected in the volatile profiles of ‘BQ-F' and ‘DK-F', but were absent in ‘XZH-F'. In contrast, β-elemene was uniquely present in ‘DK-F', indicating cultivar-specific differences in sesquiterpene composition.

Regarding ester components, isobornyl formate stood out with relatively consistent levels across all varieties; highest in ‘BQ-F' (1.90 ± 0.06%), followed by ‘DK-F' (1.85 ± 0.08%) and ‘XZH-F' (1.81 ± 0.07%). Benzoic acid esters, including methyl and ethyl esters, were detected at low levels, but there was varietal variation. Benzoic acid, ethyl ester was most abundant in ‘BQ-F' (0.17 ± 0.02%), with significantly lower levels in ‘DK-F' (0.02 ± 0.00%) and ‘XZH-F' (0.04 ± 0.00%), suggesting a unique aromatic contribution to ‘BQ-F'. Heptanoic acid, ethyl ester was identified in ‘BQ-F' and ‘XZH-F' but was absent in ‘DK-F'. In contrast, dodecanoic acid, ethyl ester was exclusively detected in ‘DK-F', highlighting distinct ester profiles among the cultivars.

α-2-Propenyl-benzyl alcohol was the predominant alcohol compound, with the highest accumulation in ‘BQ-F' (1.20 ± 0.04%), followed by ‘DK-F' (1.17 ± 0.05%) and ‘XZH-F' (1.15 ± 0.05%). In contrast, benzenemethanol, 4-hydroxy, and 1,2-octanediol were absent in ‘XZH-F' but present in both ‘BQ-F' and ‘DK-F'. 2-Nonenal was the most abundant aldehyde detected in the distilled liquor of all three varieties, while trans-cinnamic acid was exclusively present in ‘BQ-F' and absent in both ‘DK-F' and ‘XZH-F'. Overall, the data indicate that ‘BQ-F' has a more complex and intense volatile profile than ‘DK-F' and ‘XZH-F', particularly in terms of terpenoid and ester content. These differences could influence sensory perception and consumer preferences of fermented juices.

### 3.6 Odor activity analysis and key aroma-active compounds in fermented juice

To directly assess which volatiles genuinely contribute to the aroma of fermented juice, odor activity analysis was integrated with the metabolomic profiling data. A total of 293 odor-active compounds were identified in the top ten aroma descriptor categories in the three fermented juice varieties **(Fig. S5A)**. The most highly represented categories were fruity (43 compounds), green (43), and sweet (42), followed by woody (41), herbal (28), floral (25), spicy (19), citrus (18), waxy (18), and fresh (16) **(Fig. S5B)**. Among the compounds with documented odor thresholds, several had exceptionally low values, indicating the highest aroma impact potential **(Table S5)**.

The fruity character was dominated by 3-mercaptohexyl acetate (threshold: 0.00002 μg/L) and 3-mercaptohexanol (threshold: 0.00006 μg/L), two sulfur-containing compounds with extraordinarily potent tropical-fruity notes even at trace concentrations. The green notes were primarily attributable to 2-Nonenal, (E) (threshold: 0.0001 μg/L) and 6-Nonenal, (Z) (threshold: 0.00014 μg/L), unsaturated aldehydes generated through lipoxygenase-mediated fatty acid oxidation. The cross-category compound 3,5-Octadien-2-one, (E, E) (threshold: 0.0005 μg/L) emerged as a key contributor to both fruity and green descriptors. Sweet and floral attributes were strongly linked to 2H-Pyran, tetrahydro-4-methyl-2-(2-methyl-1-propenyl) (threshold: 0.0002 μg/L) and α-ionone (threshold: 0.00378 μg/L), both terpenoid-derived compounds. Terpenoids were the dominant compound class across all ten aroma categories (111 of 293 compounds), followed by esters (91) and aldehydes (32), consistent with the metabolomic profiling data and confirming their central role in shaping the overall aroma architecture of fermented bayberry juice.

### 3.7 Comparative analysis of aroma components in the distilled liquor of three bayberry varieties

**Fig. 4:**
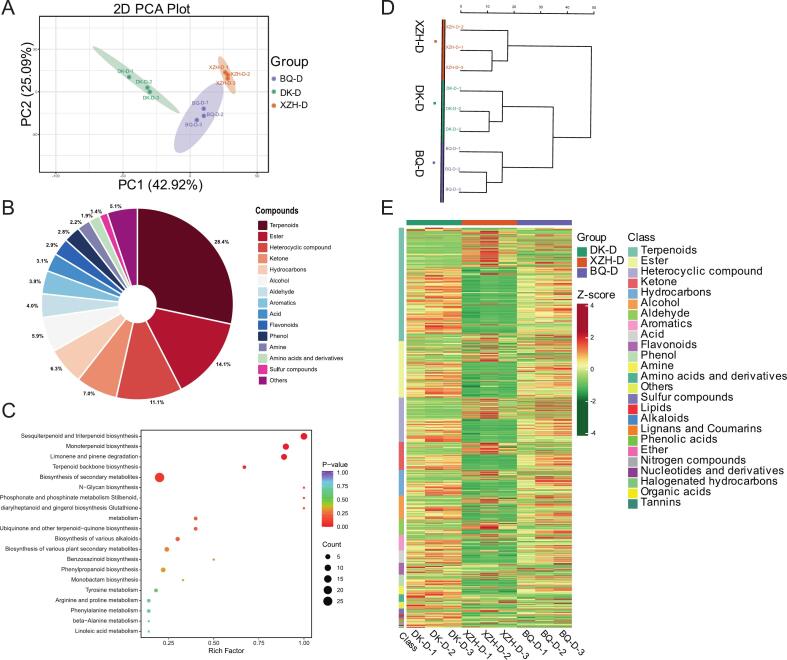
Overview of metabolites and aroma compounds comparing three bayberry varieties (‘Biqi’, ‘Dongkui’, and ‘Xiazhihong’) at the distilled liquor stage. (A) Principal component analysis (PCA). (B) Pie chart depicting the percentage of alteration in metabolite categories. (C) KEGG enrichment analysis. (D) Hierarchical clustering analysis of the three varieties. (E) Heatmap for the three bayberry varieties, with color indicating metabolite accumulation level, ranging from low (green) to high (red). (For interpretation of the references to color in this figure legend, the reader is referred to the web version of this article.)

The comparative analysis of distilled bayberry liquors from three varieties revealed significant compositional differences, as demonstrated by integrated metabolomic approaches **(**Fig. 4**)**. PCA showed distinct clustering patterns of varieties, with PC1 (42.92%) and PC2 (25.09%) collectively explaining the total variance **(**Fig. 4A**)**. The PCA plot revealed tight intra-variety clustering with clear separation between groups, particularly along PC2 where ‘XZH-D' samples formed a distinct cluster from ‘BQ-D' and ‘XZH-D', suggesting fundamental biochemical differences in their distilled products. Compositional analysis **(**Fig. 4B**)** identified terpenoids as the dominant chemical class in all distilled liquors (28.4% of total volatiles), followed by esters (14.1%) and heterocyclic compounds (11.1%). Comparing the three varieties, KEGG enrichment analysis **(**Fig. 4C**)** revealed distinct metabolic accumulation. Sesquiterpenoid and triterpenoid biosynthesis (Rich Factor = 0.95), monoterpenoid biosynthesis (Rich Factor = 0.89), and terpenoid backbone biosynthesis (Rich Factor = 0.87) align with the alteration percentage of the compositional analysis. Hierarchical clustering placed ‘DK-D' between ‘BQ-D' and ‘XZH-D', showing a closer similarity to ‘BQ-D' than to ‘XZH-D' **(**Fig. 4D**)**.

The heatmap analysis revealed distinct metabolic signatures characterized by differential accumulation of volatile and aromatic compounds **(**Fig. 4E**)**. *Z*-score-normalized data revealed pronounced varietal differences in key chemical classes, with the most significant variation in terpenoids. ‘DK-D' had the strongest positive modulation of terpenoids (Z-score: 1.3–3.5), particularly monoterpenes and sesquiterpenes. The levels were moderate in ‘BQ-D' (Z-score: 1.1–2.2), while ‘XZH-D' had lower terpenoid content (downregulated). Ester compounds, crucial for fruity aroma profiles, were predominantly enriched in ‘DK-D' and ‘BQ-D', with significantly lower levels in ‘XZH-D'. This pattern was inversely correlated with alcohol content, where ‘DK-D' had the highest alcohol levels (Z-score: 2.9) compared to ‘BQ-D' (Z-score: 1.3) and ‘XZH-D' (Z-score < 0). The results demonstrate that varietal genetic background significantly influences the final composition of distilled bayberry liquors, with each cultivar maintaining distinct chemical fingerprints throughout distillation. These findings provide a molecular basis for developing variety-specific quality standards and distillation protocols to optimize flavor profiles and bioactive compound retention in bayberry spirits.

There was a critical difference in several categories of the top differential volatile components in the distilled liquor of three varieties, especially the profiles of terpenoids and esters, which are known for their strong relation to aroma and flavor characteristics **(Table S6)**. The terpenoid profiles were distinct among the varieties, with the highest levels in ‘BQ-D', especially for β-caryophyllene (4.22 ± 0.32%) and β-cedrene (2.67 ± 0.23%), compounds known for their spicy and woody notes. ‘DK-D' had intermediate terpenoid levels, while those of ‘XZH-D' were significantly lower (*p* < 0.05), with β-caryophyllene at only 0.78 ± 0.19% and β-cedrene at 0.40 ± 0.09%. Oxygenated terpenes followed a different pattern, with higher caryophyllenyl alcohol content in ‘DK-D' (0.19 ± 0.01%) compared to other varieties. Both γ-terpinene and α-terpinene were detected in the volatile profiles of ‘BQ-D', but were absent in ‘DK-D' and ‘XZH-D'.

For ester metabolites and among the three varieties, ‘XZH-D' had the highest concentrations of ethyl caprate (14.72 ± 2.25%), ethyl 9-decenoate (13.78 ± 2.07%), and formic acid butyl ester (6.40 ± 1.18), followed by ‘DK-D' (13.49 ± 1.01%, 12.46 ± 0.98%, and 4.83 ± 0.74, respectively) and ‘BQ-D' (12.58 ± 0.84%, 11.75 ± 0.84%, and 4.86 ± 0.17). Hexanoic acid, propyl ester was absent in ‘DK-D' but present in both ‘XZH-D' (0.08 ± 0.01) and ‘BQ-D' (0.02 ± 0.00), whereas octanoic acid, methyl ester was detected only in ‘BQ-D' and ‘DK-D'. Uniquely, acetic acid, heptyl ester accumulated only in ‘XZH-D'. These differences suggest varietal specificity in ester retention or formation during distillation. Alcohol profiles further revealed varietal divergence; ‘BQ-D' (1.07 ± 0.04%) and ‘XZH-D' (1.00 ± 0.23%) had nearly double the 1-hexanol content compared to ‘DK-D' (0.42 ± 0.03%), while phenolic alcohols such as benzenemethanol, 4-hydroxy were significantly higher in ‘BQ-D' (0.98 ± 0.07%) and ‘DK-D'(0.73 ± 0.09%), approximately 4–5 times that of ‘XZH-D' (0.18 ± 0.04%). Notably, 3-hexen-1-ol (Z) was absent in ‘DK-D', and 2-nonanol was unique to ‘XZH-D'.

Ketone levels, especially 3-hexanone, 1-phenyl, were markedly lower in ‘XZH-D' (0.54 ± 0.13) compared to ‘BQ-D' (3.05 ± 0.27) and ‘DK-D' (2.31 ± 0.31). ‘XZH-D' also lacked nerylacetone and β-dihydroionone, compounds present in the other two varieties. In contrast, sulcatone was absent in ‘BQ-D'. Aldehyde profiles showed higher diformylhydrazine in ‘DK-D' (0.96 ± 0.12) and ‘XZH-D' (0.85 ± 0.03) than in ‘BQ-D' (0.58 ± 0.03). The results demonstrate that varietal origin significantly influences the final composition of distilled bayberry liquors, with each variety producing spirits with distinct chemical and likely sensory profiles. These findings provide a chemical basis for quality differentiation and suggest opportunities to optimize distillation processes and enhance desirable varietal characteristics.

### 3.8 Odor activity analysis and key aroma-active compounds in distilled liquor

**Fig. 5:**
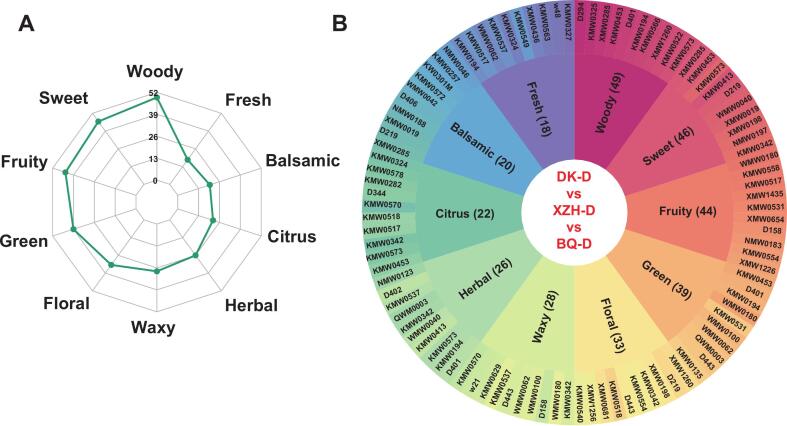
Odor activity of aroma-active compound profiles for distilled liquor from three Chinese bayberry varieties (‘Biqi’, ‘Dongkui’, and ‘Xiazhihong’). (A) Radar chart showing the number of odor-active compounds across ten aroma descriptor categories; (B) Wheel chart showing the distribution of individual odor-active compounds within each aroma category.

Odor activity analysis of the distilled liquor revealed a substantially expanded and reorganized aroma landscape compared to the fermented juice, reflecting the selective enrichment and transformation of volatile compounds during distillation. A total of 325 odor-active compounds were distributed across the top ten aroma descriptor categories in the three distilled liquor varieties **(**Fig. 5A**)**. The aroma profile shifted markedly towards woody (49 compounds), sweet (46), and fruity (44) as the three dominant categories, followed by green (39), floral (33), waxy (28), herbal (26), citrus (22), balsamic (20), and fresh (18) **(**Fig. 5B **and Table S7)**. Notably, the spicy category in fermented juice was replaced by a new balsamic category in the distilled products, indicating that distillation selectively enriched resinous and warm-aromatic compounds while suppressing some pungent volatiles. The single most aroma-potent compound across the entire distilled liquor profile was β-ionone, a terpenoid compound with an exceptionally low odor threshold of 0.000007 μg/L, which contributed simultaneously to the woody, sweet, fruity, and floral categories, making it the highest-impact aroma-active compound identified in this study.

The waxy and green characters were anchored by 2,4-Decadienal (E, E) (threshold: 0.00007 μg/L) and 6-Nonenal (Z) (threshold: 0.00014 μg/L), respectively, both unsaturated aldehydes derived from lipid oxidation. Sweet and floral notes were further reinforced by 2H-Pyran, tetrahydro-4-methyl-2-(2-methyl-1-propenyl) (threshold: 0.0002 μg/L), and 4-methoxybenzaldehyde (threshold: 0.0002 μg/L), with the latter also contributing to the balsamic character unique to the distilled products. The citrus category was distinguished by dodecanenitrile (threshold: 0.00009 μg/L), a nitrogen-containing compound not detected as a threshold-active contributor in fermented juice. As in the fermented juice, terpenoids remained the dominant compound class in the distilled liquor (115 of 325 compounds), followed by esters (91) and aldehydes (38), although the absolute number of aldehyde-class contributors increased from 32 in fermented juice to 38 in distilled liquor, consistent with the thermal generation of aldehydes during distillation. Together, the odor activity profiles of the fermented juice and distilled liquor confirm that the aroma of bayberry spirits is driven by a small number of ultra-potent terpenoids and aldehydes operating well below their odor thresholds, underpinning the importance of preserving these compounds throughout the production process.

### 3.9 Sensory evaluation of distilled liquor among bayberry varieties

Sensory evaluation of distilled bayberry liquors revealed significant varietal differences in organoleptic properties **(**Table 3**)**. While all three varieties recorded perfect scores for appearance (9.3–9.7), their aroma profiles had distinct characteristics. ‘BQ-D' had the most intense and complex aroma profile (25 ± 1), higher than ‘XZH-D' (19.1 ± 0.8, *p* < 0.05), while ‘DK-D' had intermediate scores (22 ± 2). This aligns with our chemical analyses showing the superior retention ‘Biqi’ in of terpenoids and phenolic compounds that contribute to aroma complexity. ‘BQ-D' (34 ± 2) and ‘DK-D' (34 ± 2) were superior in taste evaluation, outperforming ‘XZH-D' (30 ± 4). This taste preference correlates with the balanced acid profiles and higher ester content of both ‘Dongkui’ and ‘Biqi’ revealed from our metabolomic data. The typicality scores, representing how well each liquor expressed characteristic bayberry flavors, were highest for ‘DK-D' (17.9 ± 0.4), followed by ‘BQ-D' (17 ± 2) and ‘XZH-D' (16 ± 1). Total sensory scores showed the superiority of ‘BQ-D' (85 ± 3), followed by ‘DK-D' (83 ± 5), outperforming ‘XZH-D' (74 ± 6). These results suggest that the aroma profile of ‘Biqi’ may elicit more diverse subjective responses, possibly due to its more complex aroma composition. The sensory results complement our chemical analyses and provide consumer-relevant validation of the varietal differences in distilled bayberry products.

**Table 3:** Sensory evaluation of Chinese bayberry distilled liquor.

**Indexes**	**Biqi**	**Dongkui**	**Xiazhihong**
Appearance	9.7 ± 0.4 **a**	9.4 ± 0.9 **a**	9.3 ± 0.4 **a**
Aroma	25 ± 1 **a**	22 ± 2 **b**	19.1 ± 0.8 **c**
Taste	34 ± 2 **a**	34 ± 2 **a**	30 ± 4 **b**
Typicality	17 ± 2 **a**	17.9 ± 0.4 **a**	16 ± 1 **a**
**Total**	**85 ± 3 a**	**83 ± 5 a**	**74 ± 6 b**

Values are means ± SD (*n* = 10 trained panelists). Different lowercase letters within the same row indicate statistically significant differences between varieties (one-way ANOVA, Tukey HSD post-hoc test, p < 0.05). Numerical values are rounded (SD rounded to 1 significant figure; mean rounded to the same decimal place as rounded SD).

### 3.10 Correlation between key volatile compounds and sensory scores in ‘Biqi’ distilled liquor

‘BQ-D' had the highest total sensory score among the three varieties (85 ± 3; Table 3), so it was selected for the Mantel test correlation and Pearson correlation analysis to identify the specific volatile compounds responsible for its superior distilled sensory performance. Correlations were computed between the relative contents of 30 key volatile compounds (16 terpenoids and 14 esters) and the two significant sensory attributes (aroma and taste) (**Fig. S6** and **Table S8**). Among the terpenoids, cedrene (*r* = 0.999), (−)-aristolene (*r* = 0.998), α-humulene (*r* = 0.991), β-elemen (*r* = 0.989), β-cedrene (*r* = 0.951), norbornane (*r* = 0.944), and longifolene (*r* = 0.929) gave strong positive correlations with both aroma and taste, suggesting these sesquiterpenes are important contributors to the overall sensory profile. β-gurjenene (*r* = 0.856), 3-Pinanol (*r* = 0.749), and γ-terpinene (*r* = 0.711) had moderately strong positive associations. In contrast, linalool had a strong negative correlation (*r* = −0.902), while α-terpinene (*r* = −0.793) was negatively associated with sensory quality. Among the ester compounds, butanoic acid hexyl ester (*r* = 0.982), formic acid butyl ester (*r* = 0.973), butanoic acid heptyl ester (*r* = 0.912), and propanoic acid 2-methyl- ethyl ester (*r* = 0.910), strongly and positively correlated with both sensory attributes, while benzoic acid methyl ester (r = −0.793), benzoic acid ethyl ester (*r* = −0.730), and heptanoic acid ethyl ester (*r* = −0.655) were negatively associated with sensory quality. Collectively, these correlations provide the compound-level quantitative explanation for the superior sensory performance of ‘BQ-D' and confirm that its distinctive aroma quality is not attributable to any single compound but to a characteristic terpenoid-ester balance that distinguishes it from the other two varieties.

## 4 Discussion

Aroma is a critical quality attribute of fruit juices, shaping consumer preferences. However, the evolution of aroma compounds during fermentation and distillation is a complex process, influenced by dynamic biochemical reactions, fermentation time, and the fruit matrix (Tian et al., 2024). Our multivariate analysis revealed distinct metabolic signatures across processing stages (juice, fermented, and distilled) and clear varietal differentiation, highlighting significant compositional differences attributable to both cultivar characteristics and processing effects **(Fig. S7)**.

### 4.1 Terpenoid biosynthesis and degradation during processing

Our comparative metabolomic analysis of three bayberry varieties revealed substantial alterations in volatile profiles, primarily affecting terpenoids and esters, the two dominant aroma-contributing classes **(**Fig. 1G-H **and Fig. S3G—H)**. Terpenoids, known for imparting “woody”, “floral”, and “herbal” notes, are recognized as signature aroma components of bayberry (Mahanta et al., 2021; Saeed et al., 2025). Previous reports have indicated that terpenes account for 59.9% of the total volatile content in bayberry fruit and 24.5% in the juice (Zhang et al., 2024).

KEGG enrichment analysis revealed significant perturbations in terpenoid backbone biosynthesis pathways. In plants, the mevalonate (MVA) pathway in the cytoplasm produces sesquiterpenes and triterpenes, while the methylerythritol phosphate (MEP) pathway in plastids generates monoterpenes and diterpenes (Hu et al., 2025). These pathways converge to produce isopentenyl diphosphate and dimethylallyl diphosphate, which serve as universal five‑carbon precursors for all terpenoid biosynthesis (Guo et al., 2025; Kumari et al., 2024). Our results demonstrate substantial reductions in key terpenes, including β-caryophyllene, linalool, and β-elemene, during processing **(Tables S4 and S6)**. There was a significant reduction of β-Caryophyllene, a sesquiterpene derived from farnesyl diphosphate, in all varieties, particularly in ‘XZH-D', likely due to its susceptibility to oxidative degradation and thermal isomerization during processing (Cheng et al., 2025). The retention of more diverse terpene profiles in ‘BQ-F' and ‘DK-F', including cedrene, β-cedrene, α-curcumene, and caryophyllenyl alcohol, suggests cultivar-specific differences in terpene synthase expression or endogenous antioxidant capacity (Cheng, Chen, Chen et al., 2015). Linalool degradation into nerol, α-terpineol, and geraniol through acid-catalyzed hydration represents a loss of the original floral-citrus character while generating new aroma-active monoterpene alcohols (Cheng et al., 2016; Kang et al., 2012). The decline of β-elemene and calarene, associated with “sweet” aromas, further underscores aroma degradation during juice processing (Zhang et al., 2024).

While KEGG enrichment of terpenoid biosynthesis pathways was consistent with the observed compound-class changes, it should be noted that metabolite data alone provide associative rather than mechanistic evidence. Terpenoid accumulation is regulated by key enzymes in the MEP and MVA pathways, including 1-deoxy-D-xylulose 5-phosphate synthase (DXS), 1-deoxy-D-xylulose 5-phosphate reductoisomerase (DXR), 3-hydroxy-3-methylglutaryl-CoA reductase (HMGR), and terpene synthases (TPS) (Hu et al., 2025). Previous studies have shown that sesquiterpene biosynthesis is closely linked to the MVA pathway gene expression, while TPS activity is further controlled by transcription factors such as MYB, bHLH, and WRKY (Hu et al., 2025; Li et al., 2023). The cultivar-dependent differences observed here may therefore reflect variation in TPS expression. In addition, fermentation-related microbial activity and enzymatic transformations likely contribute to terpenoid diversification. Overall, the observed changes suggest potential shifts in MEP/MVA pathway activity and require further validation through transcriptomic or enzymatic analyses.

### 4.2 Ester biosynthesis and fatty acid metabolism

During processing, there were equally important transformations of esters, crucial contributors to fruity, floral, and sweet sensory attributes. KEGG pathway enrichment highlighted fatty acid degradation and biosynthesis as pivotal metabolic processes. Lipoxygenase-mediated oxidation of linoleic and linolenic acids initiates the formation of volatile precursors, which undergo subsequent β-oxidation to yield medium-chain fatty acids. These acids serve as substrates for alcohol acyltransferase (AAT)-catalyzed esterification reactions with ethanol and higher alcohols generated through yeast fermentative metabolism. AATs belong to the BAHD superfamily and catalyze the final decisive step in ester formation by transferring an acyl group from acyl-CoA to an alcohol acceptor (Liu, Huang, & Lian, 2023; Saez et al., 2024).

The overall increase in esters during fermentation is consistent with their formation from sugars and amino acid metabolism (Pu et al., 2023). Our analysis classified ethyl caprate as a vital compound, positively affected by juice processing and dependent on variety. Capric acid accumulates through β-oxidation of longer-chain fatty acids, subsequently undergoing AAT-mediated esterification with ethanol to yield ethyl caprate, imparting desirable fruity, peach-like, and almond notes. This aligns with previous observations in hawthorn wines and grapefruit (Tian et al., 2024; Wei et al., 2022). The varietal dependence of ester profiles suggests genotype-specific differences in substrate availability and enzymatic activity.

While metabolite-level data implicate fatty acid degradation and AAT-mediated esterification in ester enrichment during bayberry processing, this inference requires transcriptomic validation, as the full lipoxygenase (LOX) to hydroperoxide lyase to alcohol dehydrogenase (ADH) to AAT cascade is entirely gene-expression-dependent at each step (Yu et al., 2025; Zhang et al., 2020). In the Korla pear, transcriptomic analysis identified *PsLOXL*, *PsADHL*, and *PsAATL* as key genes that directly explain elevated ester levels at maturity (Liu, Wen, et al., 2023). The variety-specific ester profiles in this study, particularly the elevated levels of ethyl caprate and ethyl 9-decenoate in distilled liquor, most likely reflect cultivar-dependent differences in AAT gene expression or substrate specificity. Transcriptomic characterization of the LOX and AAT gene families in *Morella rubra* remains an essential future research priority.

### 4.3 Organic acid metabolism and Ehrlich pathway activation

The total concentration of organic acids increased during fermentation, with novel acids produced during this stage, as previously reported for hawthorn wine (Chen et al., 2023). Organic acids play critical roles as key precursors in ester formation: acetic acid, decanoic acid, and lauric acid can be converted to ethyl acetate, ethyl caprate, and ethyl laurate, respectively, compounds that help mitigate pungency and contribute to balanced fruity aroma (Wei et al., 2022). While acids are primarily derived from triglyceride hydrolysis, lipid oxidation, or transformation of aldehydes and ketones, their direct contribution to aroma is limited due to relatively high odor thresholds (200–5000 μg/kg) (Chen et al., 2023).

Alcohols, key contributors to floral and fruity aromas, are by-products of sugar and amino acid metabolism. The significant decrease in amino acids (*p* < 0.01) post-fermentation implicates the Ehrlich pathway as a central metabolic route connecting amino acid catabolism to aroma formation. KEGG enrichment analysis corroborated the importance of amino acid degradation pathways. During fermentation, yeasts catabolize amino acids via the Ehrlich pathway: transamination converts amino acids to α-keto acids, followed by decarboxylation to aldehydes, and reduction to higher alcohols (fusel alcohols) (Zhu et al., 2024). Our findings highlighted a notable increase in compounds such as 1-hexanol **(Table S4 and S6)**, known for its sweet, floral character, consistent with previous reports (Tian et al., 2024). Similar trends have been observed in blueberry and hawthorn wines, where sweet amino acids, such as threonine, decreased by over 40% during fermentation, contributing to enhanced “floral” and “sweet” attributes (Wang, Chenhao, et al., 2023; Zhong et al., 2021). The hydrolysis of bitter amino acids during fermentation likely contributes to overall taste improvement, reducing bitterness and enhancing flavor harmony (Tian et al., 2024).

### 4.4 Aldehyde formation and degradation: Lipoxygenase pathway dynamics

Critical changes were observed in aldehyde metabolite profiles across processing stages **(Tables S4 and S6)**. Aldehydes are primarily generated through the LOX pathway, where LOX catalyzes the oxygenation of polyunsaturated fatty acids to form hydroperoxides, which are subsequently cleaved by hydroperoxide lyase to yield C6 and C9 aldehydes, including hexanal, (E)-2-hexenal, nonanal, and (E)-2-nonenal (Mathavaraj et al., 2025; Viswanath et al., 2020). Aldehyde compounds, despite their typically low concentrations, can exert a profound influence on aroma due to their low odor thresholds (Yu et al., 2020). Compounds such as nonanal and octanal contribute “fruity” and “green” notes, while (E)-2-nonenal and hexanal provide “cucumber” and “green leafy” nuances (Zhang et al., 2024).

The substantial loss of hexanal and related C6 aldehydes during juice processing represents a critical quality challenge. These volatile aldehydes undergo rapid oxidation to corresponding acids or reduction to alcohols via alcohol dehydrogenase activity. The thermal inactivation of LOX during juice extraction halts de novo aldehyde biosynthesis. In contrast, residual aldehyde dehydrogenase and oxidase activities continue to degrade existing aldehydes, resulting in net losses that diminish the characteristic “green” aroma of bayberry (Cheng et al., 2015, Zhang et al., 2024). These compounds are major targets for quality preservation in bayberry products. In summary, this study offers clear mechanistic insight into how bayberry aroma develops and changes during processing. Future research should aim to optimize fermentation conditions, such as yeast selection and temperature control, to enhance desirable esters while reducing terpene loss. Exploring the regulation of key enzymes, including terpene synthases, alcohol acyltransferases, and lipoxygenases, may also support breeding or biotechnological approaches to improve aroma quality in bayberry products.

## 5 Conclusion

This study demonstrates that the final aroma profile of Chinese bayberry liquor is decisively shaped by the interplay between varietal characteristics and processing stages, highlighting the significant metabolic transformations that occur during fermentation and distillation. Several volatile compounds, including α-phellandrene-8-ol, carveol II, norborane, isobornyl formate, and dodecanoic acid ethyl ester, were identified in bayberry in the present study and, to the best of our knowledge, have not been previously reported in the literature. Moreover, key biochemical pathways, specifically fatty acid degradation and terpenoid biosynthesis, were associated with the formation of critical aroma-active esters and terpenes. Odor activity analysis identified 293 and 325 odor-active compounds in ten aroma descriptor categories in fermented juice and distilled liquor, respectively, with β-ionone (threshold: 0.000007 μg/L) emerging as the most potent aroma contributor in the distilled product. Among the tested cultivars, the ‘Biqi’ variety had superior sensory performance, which was closely related to its distinctive volatile compositions, underscoring that genotype plays a critical role in determining final product quality. These findings provide valuable insights into the biochemical basis of bayberry flavor development and offer a scientific foundation for variety selection and process optimization in bayberry beverage production. Our research provides a practical framework for enhancing the sensory quality of bayberry liquors through strategic variety selection and controlled processing, grounded in an understanding of the underlying flavor chemistry.

## CRediT authorship contribution statement

**Mostafa Saeed:** Writing – review & editing, Writing – original draft, Visualization, Software, Methodology, Investigation, Formal analysis, Data curation, Conceptualization. **Chaochao Zhou:** Methodology, Formal analysis, Data curation. **Zhuyun Chen:** Methodology, Formal analysis, Data curation. **Guoyun Wang:** Resources, Methodology, Data curation. **Ting Tu:** Resources, Methodology, Data curation. **Huimin Jia:** Writing – review & editing. **Yun Jiao:** Writing – review & editing. **Junbei Ni:** Writing – review & editing. **Xian Li:** Writing – review & editing. **Zhongshan Gao:** Writing – review & editing, Supervision, Resources, Conceptualization. **Lan Zhao:** Writing – review & editing, Methodology, Investigation.

## Funding

This work was supported by the Ministry of Agriculture and Rural Affairs, Protection of Geographical Indication of Chinese bayberry in Yuyao, China (Grant Number: 2019–169), the Modern Seed Industry Special Project in Ningbo, China (Grant Number: 2021Z008), and Innovation and Development of Horticulture Discipline, China (Project Number: B231220.005-25, Funding Organization: Zhejiang University).

## Declaration of competing interest

The authors declare that they have no known competing financial interests or personal relationships that could have appeared to influence the work reported in this paper.

## Data Availability

Data will be made available on request.
