# Supplementary material for: Evolution of metabolite and volatile compounds in Chinese bayberry during juice processing, fermentation, and distillation
Source: Food Chem X. 2026 Jun 15;37:104108. doi: 10.1016/j.fochx.2026.104108 (PMC13312553; doi:10.1016/j.fochx.2026.104108)
Supplement: Supplementary file 1 — Supplementary material 1 [file mmc1.pdf]

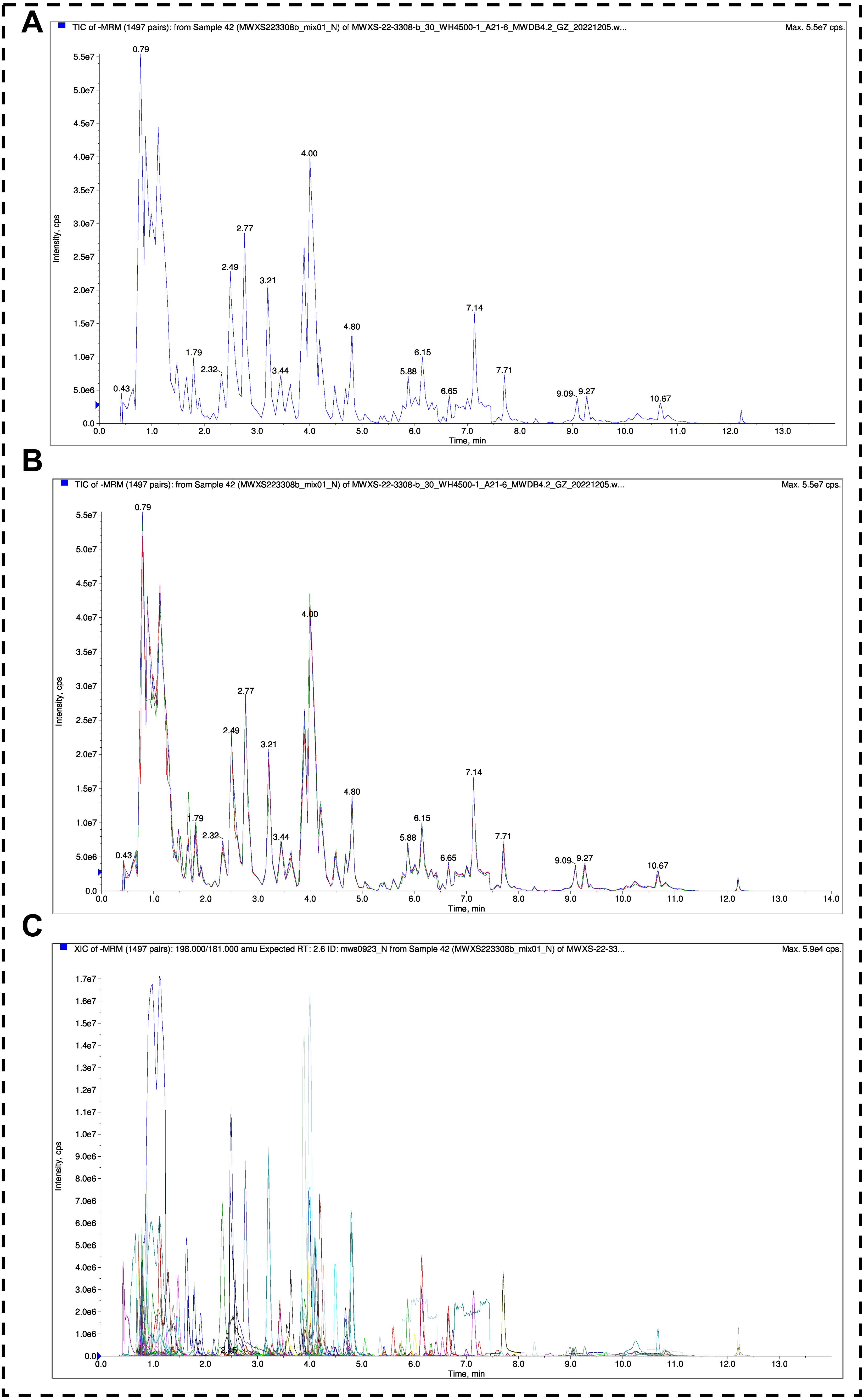

Figure S1. Representative metabolite detection and quantification samples obtained from the LC-MS platform. (A) Total ion chromatogram (TIC) of the mixed samples in negative ion mode. (B) Overlap plot of TIC from sample mass spectrometry analysis. (C) Multiple reaction monitoring (MRM) chromatograms of metabolites detected in negative ion mode.

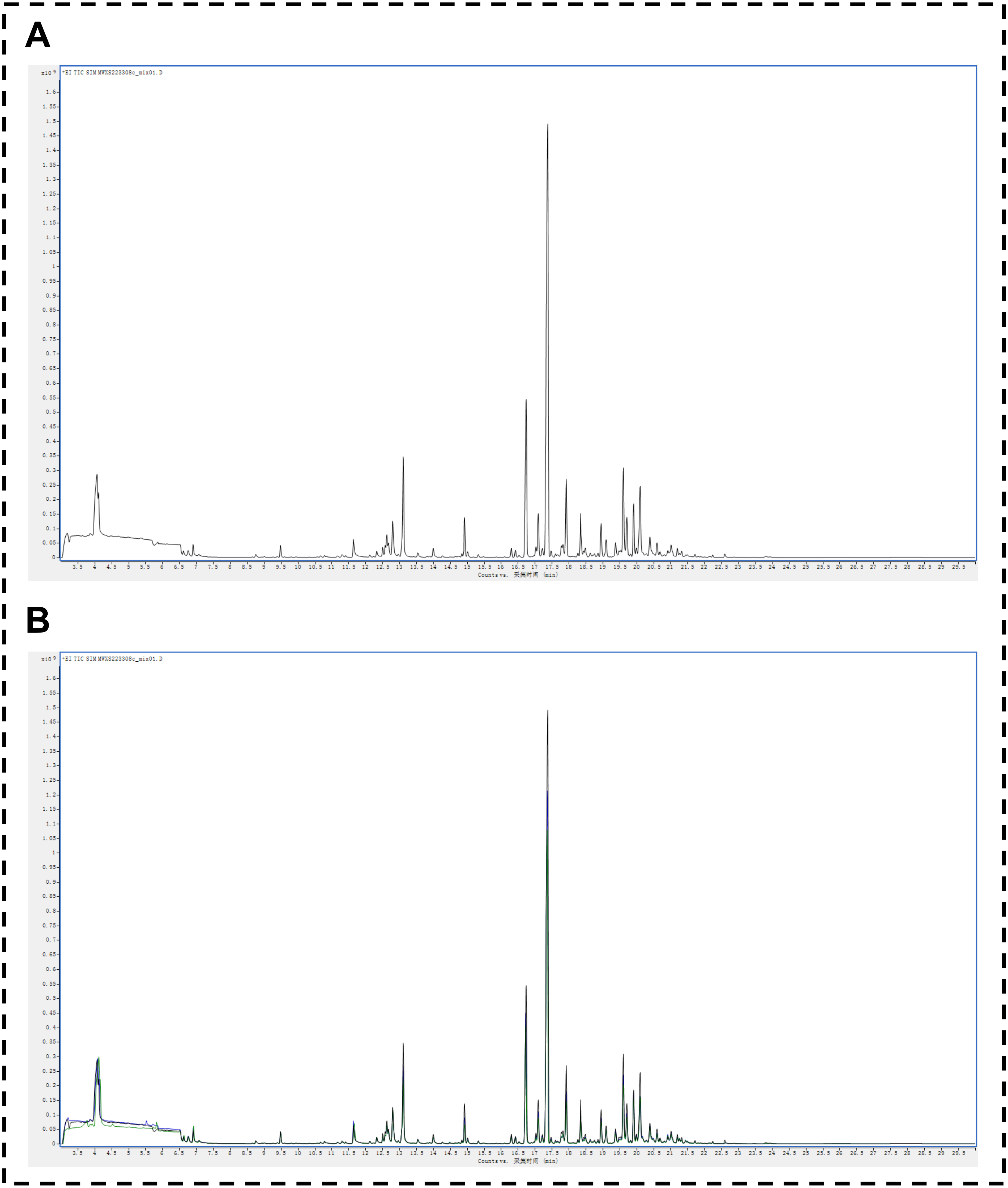

Figure S2. Representative metabolite detection and quantification samples obtained from the GC-MS platform. (A) Total ion chromatogram (TIC) for the samples. (B) Overlap plot of TIC for the samples.

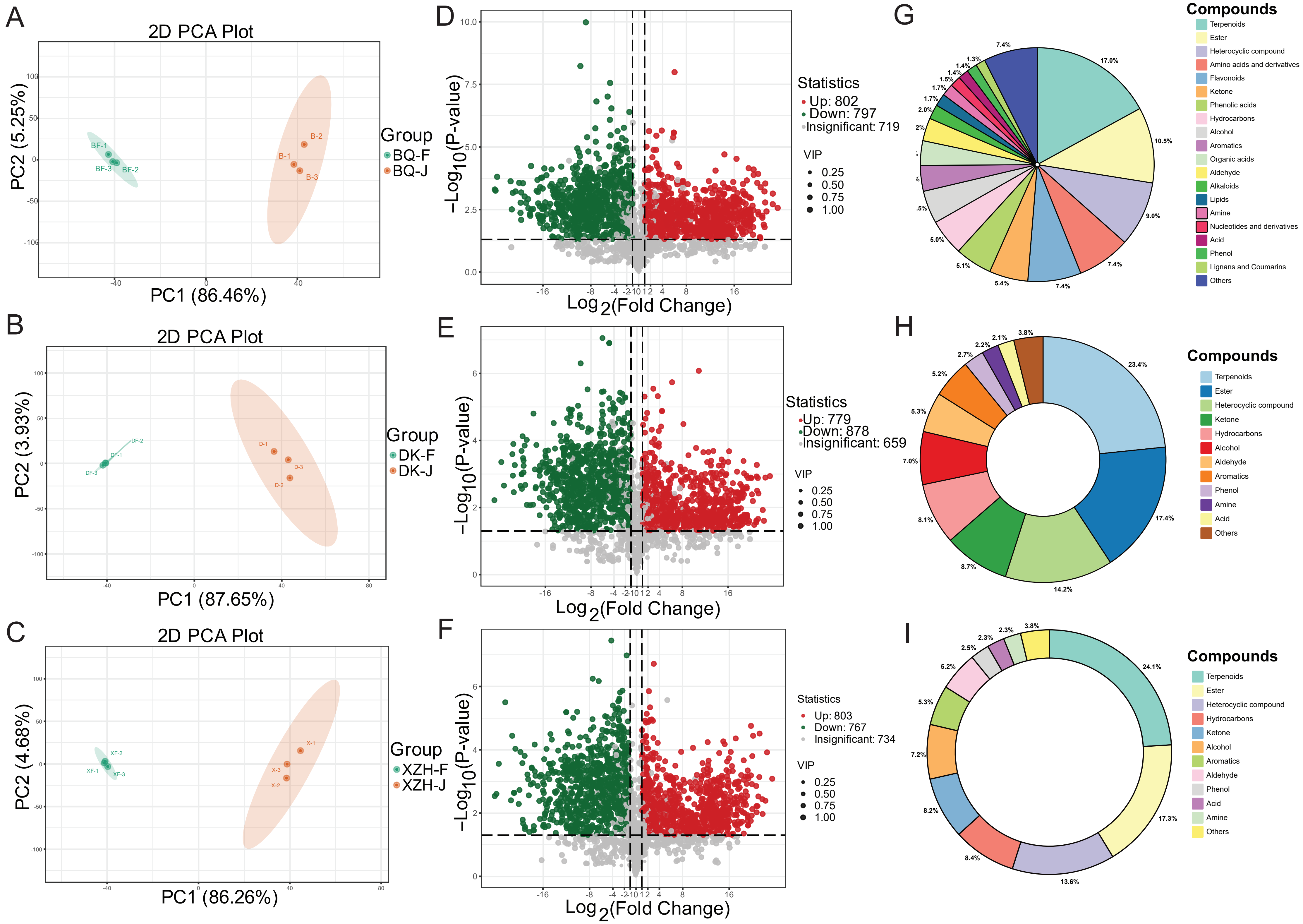

Fig. S3. Metabolomic profiling of the fermented juice compared to the fresh juice of three bayberry varieties (‘Biqi’, ‘Dongkui’, and ‘Xiazhihong’). Principal component analysis (PCA) of fermented juice vs fresh juice for ‘Biqi’ (A), ‘Dongkui’ (B), and ‘Xiazhihong’ (C). BQ-F: ‘Biqi’ fermented juice, DK-F: ‘Dongkui’ fermented juice, XZH-F: ‘Xiazhihong’ fermented juice. BQ-J: ‘Biqi’ fresh juice, DK-J: ‘Dongkui’ fresh juice, XZH-J: ‘Xiazhihong’ fresh juice. Volcano plots illustrate differentially accumulated metabolites (DAMs) in fermented and fresh juice for ‘Biqi’ (D), ‘Dongkui’ (E), and ‘Xiazhihong’ (F). The Y-axis indicates –Log10 (P-value), and the X-axis the Log2 fold change. Significantly upregulated and downregulated metabolites are highlighted in red and green, respectively, while nonsignificant compounds are shown in grey. Pie charts (G-I) display the proportional changes in metabolite classes for ‘Biqi’, ‘Dongkui’, and ‘Xiazhihong’, respectively

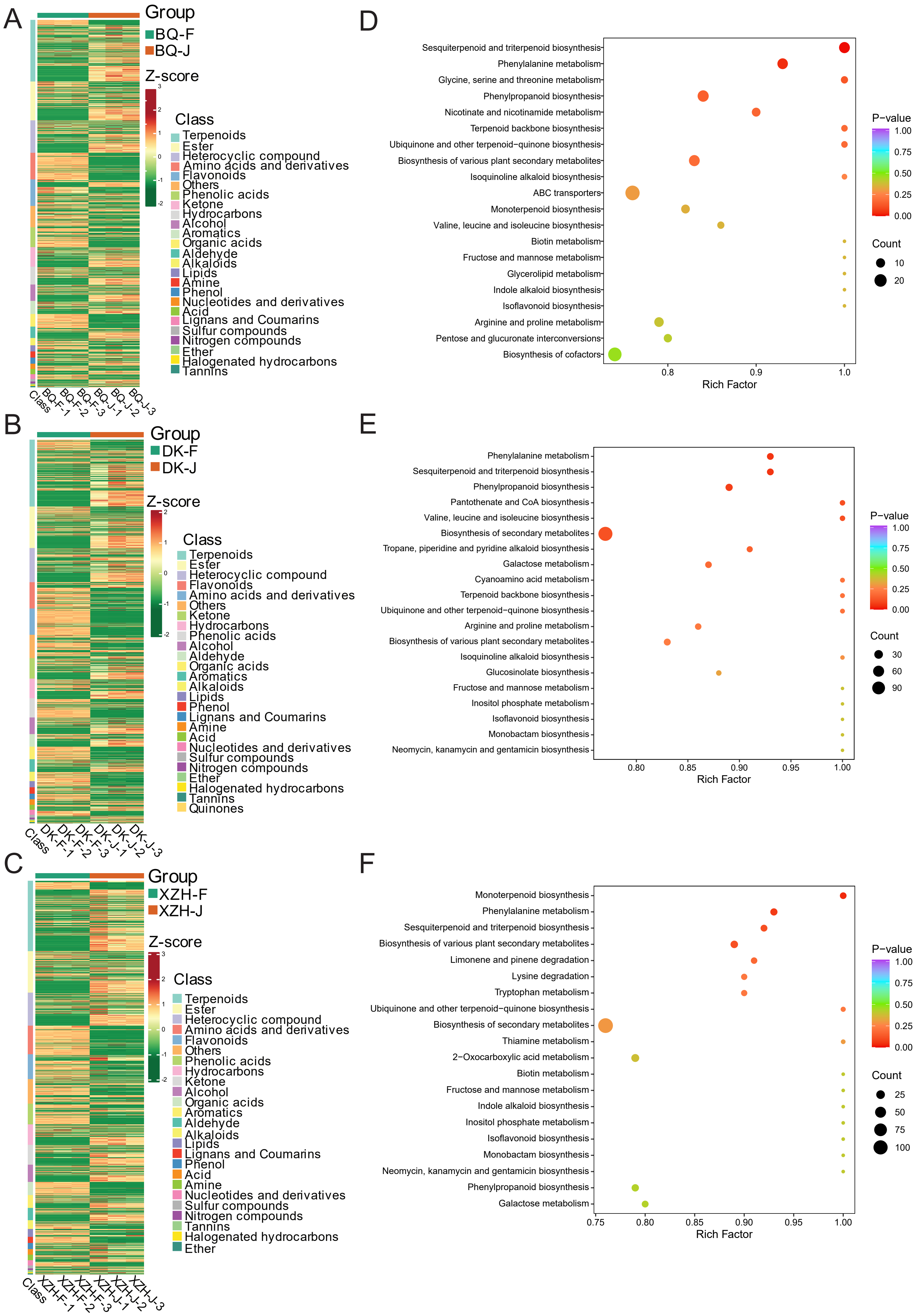

Fig. S4. Differential metabolite and KEGG-enriched pathways of three bayberry varieties at juice processing stages (fermented juice vs fresh juice). Heat map analysis of metabolites of fermented juice vs fresh juice for ‘Biqi’ (A), ‘Dongkui’ (B), and ‘Xiazhihong’ (C), with color indicating metabolite accumulation level, ranging from low (green) to high (red). The top 20 most enriched KEGG pathways of the DAMs between fermented and fresh juice for ‘Biqi’ (D), ‘Dongkui’ (E), and ‘Xiazhihong’ (F).

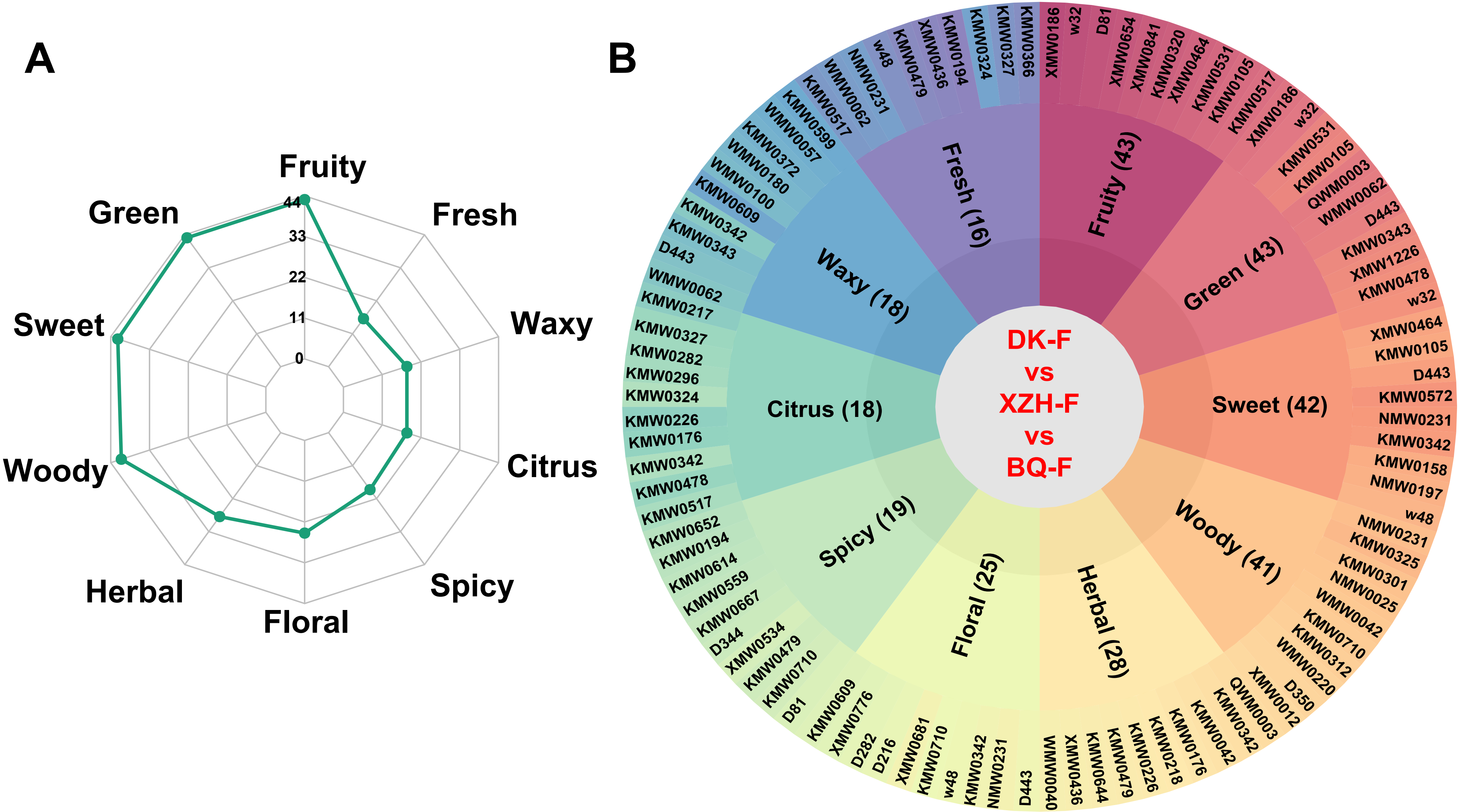

Fig. S5. Odor activity of aroma-active compound profiles for the fermented juice from three Chinese bayberry varieties ('Biqi', 'Dongkui', and 'Xiazhihong'). (A) Radar chart showing the number of odor-active compounds across ten aroma descriptor categories; (B) Wheel chart displaying the distribution of individual odor-active compounds within each aroma category.

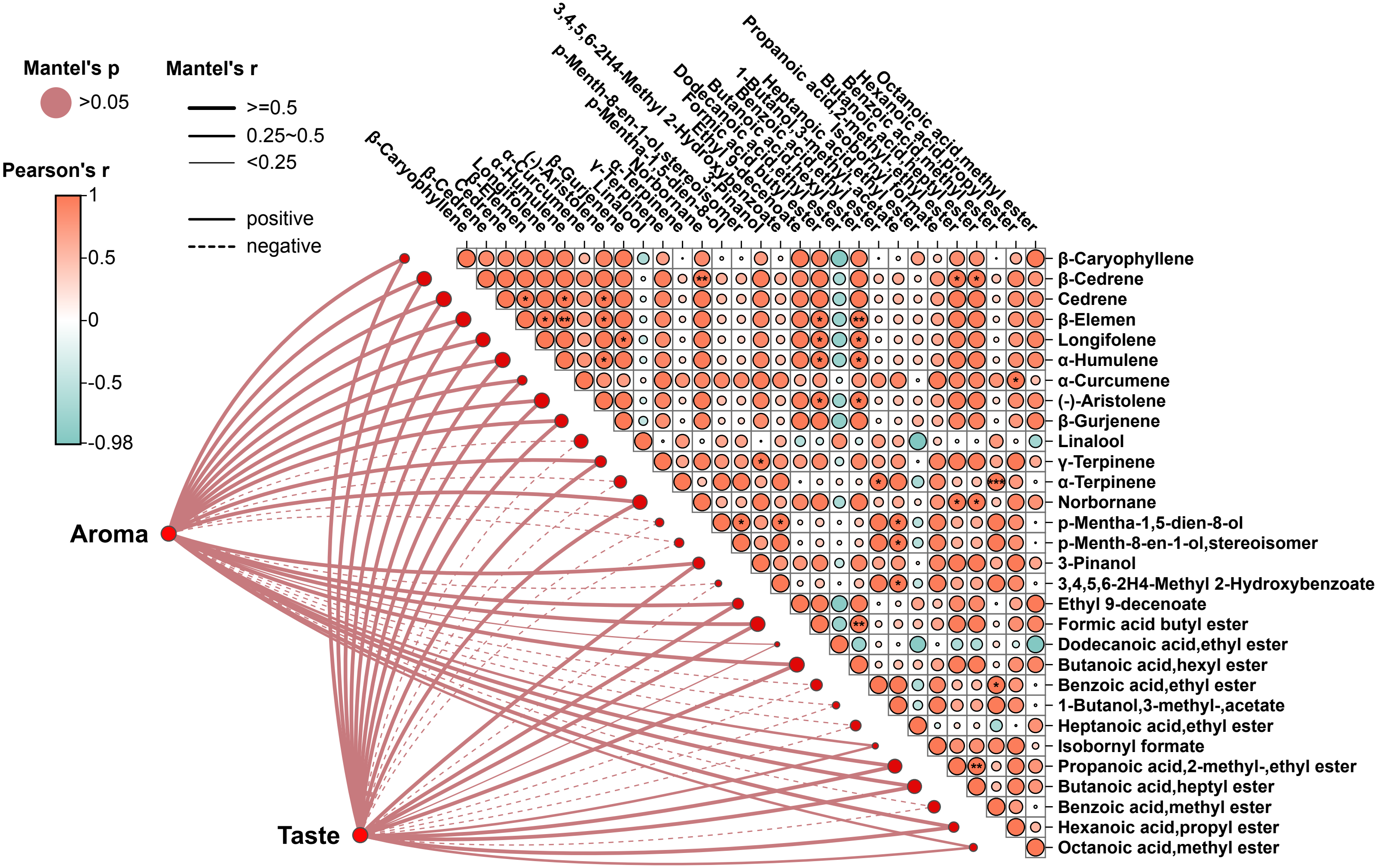

Fig. S6. Correlation analysis between sensory attributes (aroma and taste) and key volatile compounds of 'Biqi' distilled liquor based on Mantel test and Pearson's correlation.

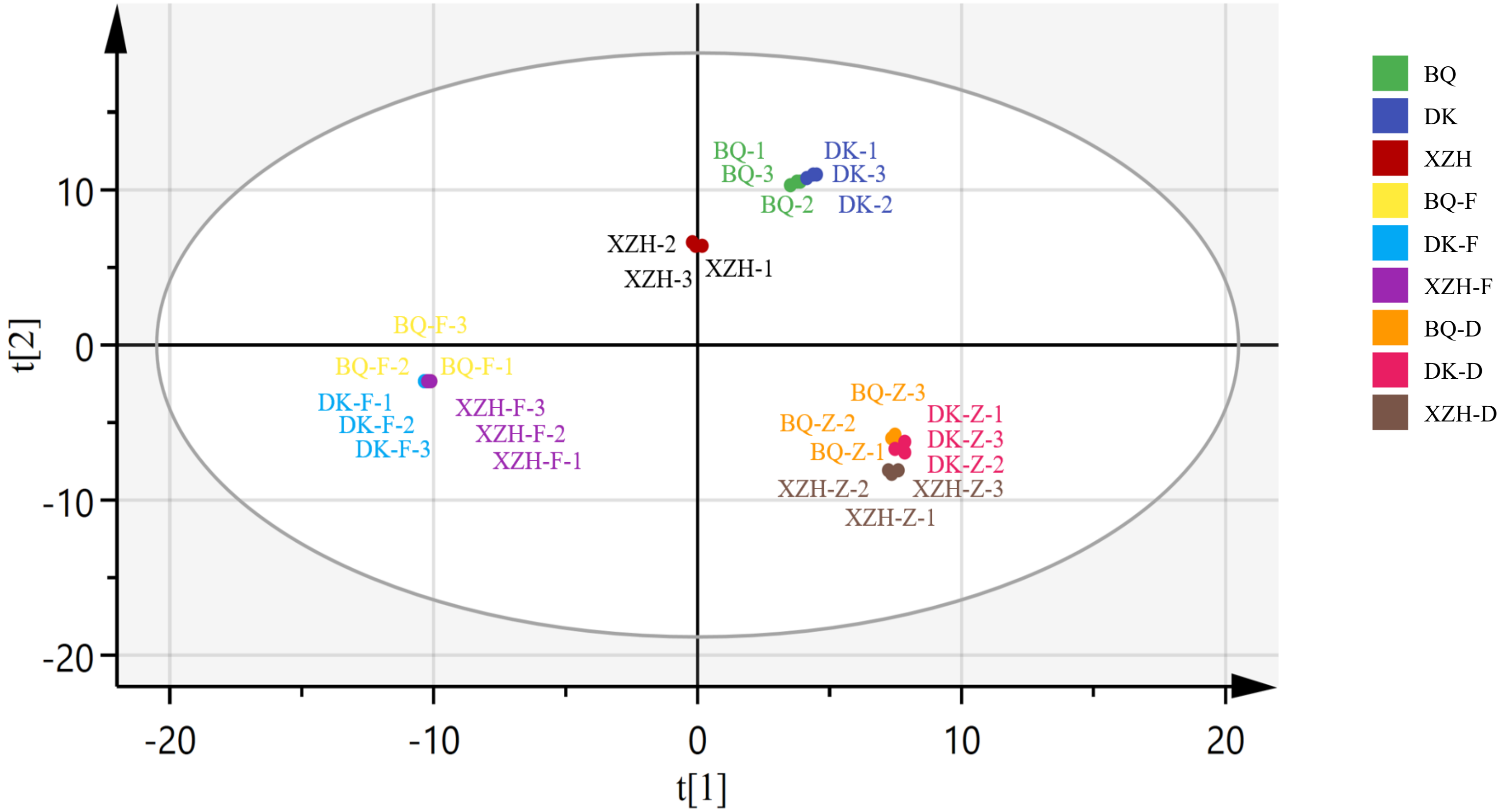

Fig. S7. OPLS-DA score plots differentiating three Chinese bayberry varieties across juice, fermented, and distilled processing stages
